# Nephroprotective and Gastroprotective potential of *Aerva javanica* (L.) green synthesized nanoparticles, supported by Molecular Networking

**DOI:** 10.1371/journal.pone.0358843

**Published:** 2026-11-05

**Authors:** Hany G. Attia, Dalia H. Abu-Baih, Bassam S. M. Al kazman, A. Maqboul, Mohamed A. Rabeh, Saad Ali Alshehri, Ali Al-shabi, Mohamed A. Zarka, Sameh S. Zaghlool, Fatma Alzahraa Mokhtar, Nehad M. Reda Abdel-maqsoud, Usama Ramadan Abdelmohsen, Khayrya A. Youssif

**Affiliations:** 1 Department of Pharmacognosy, College of Pharmacy, Najran University, Najran, Saudi Arabia; 2 Health Research Center, Najran University, Najran, Saudi Arabia; 3 Department of Biochemistry & Molecular Biology, Faculty of Pharmacy, Deraya University, Minia, Egypt; 4 Department of Clinical Pharmacy, College of Pharmacy, Najran University, Najran, Saudi Arabia; 5 Department of Pharmacognosy, College of Pharmacy, King Khalid, Abha, Saudi Arabia; 6 Department of Pharmacognosy and Phytochemistry, College of Pharmacy, The Islamic University, Najaf, Iraq; 7 Department of Pharmacology and Toxicology, Faculty of Pharmacy, Modern University for Technology and Information (MTI), Mokattam, Cairo, Egypt; 8 Department of Pharmacognosy, Faculty of Pharmacy, El Saleheya El Gadida university, Sharkia, Egypt; 9 Department of Pathology, Deraya University, Universities Zone, New Minia City, Egypt; 10 Department of Pharmacognosy, Faculty of Pharmacy, Minia University, Minia, Egypt; 11 Department of Pharmacognosy, Faculty of Pharmacy, Deraya University, Minia, Egypt; Priyadarshini JL College of Pharmacy, INDIA

## Abstract

NSAIDs pose a significant risk to the stomach and kidneys, with oxidative stress being a key driver in the development of indomethacin-induced gastric ulcers and acute kidney injury by disrupting the balance between oxidation and antioxidation. Effective management of oxidative stress and bolstering antioxidant defenses are crucial strategies for preventing and treating these conditions. This study explored the potential of Aerva javanica, a plant abundant in Saudi Arabia and used in traditional medicine, to mitigate these adverse effects. Researchers identified 27 phytochemicals in its leaf extract using LC-MS and synthesized silver nanoparticles (SNPs), characterizing them with TEM and UV-visible spectrophotometry. Network analysis suggested a link between procyanidin dimer and kaempferol 4’-O-methyl ether with genes protecting the kidneys and stomach, possibly involving the AGE-RAGE and HIF-1 signaling pathways. In vivo experiments using an indomethacin-induced injury model demonstrated that administering *A. javanica* resulted in a dose-dependent decrease in gastric ulcer severity and a return of elevated serum renal markers to normal levels. Furthermore, the treatment reduced oxidative stress markers (MDA) and increased antioxidant levels (GSH) in both the stomach and kidneys. *A. javanica* also suppressed the expression of pro-inflammatory markers and inhibited the NF-κB p65 and STAT-3 signaling pathways. Histological examination corroborated these protective effects. The observed scavenging activity of A. javanica is attributed to its rich content of flavonoids and phenolic compounds. These findings suggest that A. javanica offers promising gastroprotective and nephroprotective properties against NSAID-induced complications by reducing oxidative stress and inflammation through the coordinated regulation of key signaling pathways.

## 1. Introduction

Non-steroidal anti-inflammatory medicines (NSAIDs) are commonly prescribed as analgesics, antipyretics, and anti-inflammatory agents [1]. Despite their therapeutic effectiveness, some drugs have significant adverse effects, which limit their usage. The most worrying of these is toxicity to the kidneys and digestive system [2–4]. It is commonly known that NSAIDs mediate their therapeutic actions by inhibiting cyclooxygenase (COX), an enzyme essential to prostaglandin production [5]. Decreased physiological synthesis of prostanoids has also been substantially implicated in NSAID-induced gastrointestinal and renal toxicity [6]. Prostaglandins help the kidneys maintain renal blood flow and the glomerular filtration rate. Non-selective inhibition of COX by NSAIDs may result in reduced renal perfusion and consequent dysfunction, especially under conditions of compromised effective renal perfusion pressure [7]. Additionally, it has been found that the gastrointestinal system is harmed by decreasing prostaglandin levels [1]. However, studies have shown that COX inhibition is not enough to fully explain how these drugs induce toxicity on their own [7]. Moreover, the detrimental effects of NSAIDs on the stomach and kidneys have been linked to oxidative stress and mitochondrial dysfunction [2,8]. Thus, mechanisms other than those directly related to prostaglandins appear to have a role in the pathophysiology of NSAID-induced damage to the kidney and stomach.

Indomethacin can lead to a disruption of the delicate balance between oxidative and antioxidative mechanisms in the gastric mucosa and renal cells. This imbalance results in an excessive generation of ROS, overwhelming the natural antioxidant defense systems of the gastric and renal cells. ROS directly attack the lipids, proteins, and DNA of gastric epithelial cells, impairing their integrity and function [8,9]. This disruption of the gastric mucosal barrier allows gastric acid and other harmful substances to penetrate the underlying tissues, provoking inflammation, tissue injury, and the formation of gastric ulcers [10,11]. Moreover, ROS can activate various inflammatory pathways and contribute to the release of pro-inflammatory mediators, further exacerbating the ulceration process [12]. Also, ROS can have detrimental effects on different components of the kidneys, including the renal tubules, glomeruli, and blood vessels, inducing inflammation, tissue injury, vasoconstriction, and dysregulated blood flow—hallmarks that accelerate acute kidney injury (AKI). Inflammatory mediators further exacerbate renal parenchymal damage. Therefore, the excessive generation of ROS is considered a key factor in the pathogenesis of indomethacin-induced gastric ulcer and acute kidney injury [13].

It is worth mentioning that the transcription factor nuclear factor kappa B (NF-B) is activated in part by ROS [14–16]. Renal and gastric inflammation have been linked to the transcription of inflammatory genes associated with NF-κB activation [2]. It has also been proposed that, through phosphorylation of IκBα, the inhibitory component of NF-κB, the STAT3 pathway may contribute to activation of the NF-κB pathway, while sustained STAT3 activation has, conversely, been associated with NF-κB activation [17].

Desert medicinal plants are essential for treating a wide range of illnesses. Due to their broad range of biological activities, greater safety rating compared to synthetic medications, and cheaper cost. The perennial herbs in the genus *Aerva*, which are members of the Amaranthaceae family, are distributed throughout the North Temperate Zone, particularly in Asia and the Mediterranean areas. *Aerva javanica* (Burm.f.) Juss. Ex-Schult. is a herb with shallow roots and dioecious blooms that [18]. Numerous *Aerva* species have been shown in recent research to possess cytoprotective, analgesic, antibacterial, antioxidant, and anti-inflammatory properties. It has historically demonstrated anti-hyperglycemic, diuretic, and demulcent properties [19]. The aerial part decoction is used as a diuretic, to treat kidney stones, rheumatism, headaches, edema, and hypertension [20,21]. Root decoction is used to treat chronic chest discomfort, diarrhea, headaches, and acne. It may also be used as a plaster on the face. For horses and camels, it is an emetic and purgative. Furthermore, an extract from the aerial portions of *Aerva javanica* is used to treat skin infections, hyperglycemia, diarrhea, and gonorrhea [18]. Leaves are ground into a paste and administered topically to cure granulation tissue, reduce inflammation, and heal wounds [22]. Polyphenols, terpenoids, flavonoids, and alkaloids were found, according to phytochemical analysis. The flowers of *A. javanica* yielded three novel acylated flavone glycosides, four new ecdysteroids, and the aervecdys teroid A-D. Pentacosane and heptacosane (3-allyl-6-methoxyphenol) from the oil of *Javanica* seeds were also the primary constituents [23]. Research also revealed that the nephroprotective efficacy of an ethanol extract from the *Aerva lanata* species was investigated in albino rats that had acute renal damage caused by gentamicin and cisplatin. The extract restored the normalcy of the histological alterations and demonstrated a dose-dependent decrease in the increased blood urea and serum creatinine [24].

It has been documented that green-synthesized nanoparticles may enhance bioavailability, stability, and biological efficacy of plant-derived phytochemicals, while minimizing chemical residues associated with conventional synthesis methods [25]. The objective of this study was to examine the nephroprotective and gastroprotective properties of *Aerva javanica’s* methanolic extract and its green-synthesized nanoparticles. Also, UPLC-MS metabolomic profiling was used to examine the phytochemical composition of the crude methanolic extracts, and then molecular networking was done to identify potential chemical compounds that may be involved in these properties.

## 2. Materials and methods

### 2.1. Plant material

In June 2019, fresh *Aerva javanica* leaves were gathered in Saudi Arabia. Senior botanist Mrs. Terris Labib, the chief expert for plant identification at the El-Orman Botanical Garden in Giza, Egypt, graciously validated the plants. After that, the fresh leaves were first cleaned with tap water, and then, until no contaminants remained, they were surface cleaned with distilled water. To eliminate moisture, the cleaned leaves were shade-dried for 20 days at room temperature. For later usage, the dried leaves were ground into a fine powder in a sanitized electric blender and kept out of direct sunlight in an airtight amber glass bottle.

### 2.2. Preparation of the extracts

Through cold maceration till exhaustion, 90% methanol was recovered from the finely powdered leaves. The entire alcoholic extract was mixed and allowed to evaporate at a temperature no higher than 40 °C while under decreased pressure.

### 2.3. Metabolomics analysis

According to Abdelmohsen et al. [26,27], using an Acquity Ultra Performance Liquid Chromatography system connected to a Synapt G2 HDMS quadrupole time-of-flight hybrid mass spectrometer (Waters Milford, USA), metabolomic profiling was performed on methanolic extracts of *Aerva javanica*. Using a linear binary solvent gradient from 0 to 100% eluent B over 6 minutes at a flow rate of 0.3 ml/min, chromatographic separation was performed on a BEH C18 column (2.1x100 mm, 1.7 um particle size: Waters Milford, USA) with a guard column (2.1x5 mm, 1.7 um particle size). Solvent A was 0.1% formic acid in water (v/v), and solvent B was acetonitrile. The column temperature was 40 °C, and the injection volume was 2 µL. MS Convert software was used to transform the raw data into distinct positive and negative ionization files. After that, the files were loaded into MZmine 2.10, a data mining program, to be used for formula prediction 11, peak picking, deconvolution, and deisotoping. The Dictionary of Natural Products 2015 was the database utilized for the identification of the compounds.

### 2.4. Synthesis of silver nanoparticles using *Aerva javanica* methanolic extract

To synthesize silver nanoparticles (AgNPs), 50 mg of *Aerva javanica* methanolic extract was dissolved in 1 ml DMSO, and 0.4 ml was added to 10 ml of 1 mM silver nitrate with 1 ml of 1 M NaOH in a water bath set at 60 °C for ten minutes.

#### 2.4.1. Characterization of the synthesized *Aerva javanica* SNPs by TEM.

On coated carbon grids (CCG), a drop of the synthesized SNPs was applied, and water was allowed to evaporate at room temperature to dry the drop. A JEOL JEM-1010 transmission electron microscope operating at 80 kV was used to create the electron micrographs at the Regional Centre of Mycology and Biotechnology (RCMB) at Al-Azhar University in Cairo, Egypt [28].

#### 2.4.2. Characterization of the synthesized *Aerva javanica* SNPs by UV-visible spectrometer and Fourier transform infrared spectroscopy (FTIR).

The UV–VIS spectra of the reaction media were measured at the Faculty of Pharmacy, Ain-Shams University, Cairo, Egypt, using a twin beam V-630 spectrophotometer, Jasco, Japan, at wavelengths ranging from 200 to 600 nm, to track the creation of SNPs. The functional group affixed to the surface of SNPs was characterized using the Bruker ALPHA II FTIR Spectrometer at the Nanotechnology Centre, British University, Cairo, Egypt [29].

### 2.5. In vivo indomethacin-induced gastrotoxicity

This study involved the inclusion of adult male Wister albino rats, aged 4−6 weeks, with a weight range of 200−250 grams. The animals were housed in conventional enclosures, ensuring aseptic conditions, and were subjected to regulated ambient temperature and natural light-dark cycles. The animals were supplied with a diet consisting of standard food and water, available to them at all times. The rats were given two weeks to acclimate to the prevailing conditions before commencing the experimental protocol. The experimental procedures were carried out in adherence to the ethical principles and guidelines governing the utilization, maintenance, and manipulation of experimental animals, as established by the Research Ethics Committee, Deraya Center for Scientific Research, Deraya University, Minia, Egypt; Approval No.: DCSR-01024–02). Care was taken to ensure minimal discomfort for the animals and to avoid any suffering during the experiment. The use and care of laboratory animals were done in strict accordance with the ARRIVE guidelines 2.0 [30,31].

#### 2.5.1. Experimental design.

A total of 90 rats were divided into 10 distinct groups using a computer-generated randomization sequence. This ensured an unbiased distribution of animals before the induction of toxicity or extract administration. Before the commencement of the experiment, the subjects underwent a period of fasting lasting 24 hours, during which they were deprived of food intake. However, they were provided unrestricted access to water throughout this period.

Group 1(6 rats) (The control group) received vehicle (0.5% CMC) (1 ml/rat/once, oral gavage).

Group 2 (6rats): received famotidine only (10 mg/kg /once, oral gavage) suspended in 1 ml of 0.5% CMC

Group 3(6 rats): received AgCL (1 ml/rat/once, oral gavage).

Group 4 (6 rats): received extract (200 mg/kg/once, oral gavage),

Group 5 (6 rats): received nano formulation (1 ml/rat/once, oral gavage).

Group 6 (12 rats): (The ulcer group) received vehicle (0.5% CMC) (1 ml/rat/once, oral gavage).

Group 7 (6 rats): (The famotidine-treated group) received famotidine (10 mg/kg /once, oral gavage) suspended in 1 ml of 0.5% CMC.

Group 8 (12 rats): (The low-dose extract-treated group) received extract (50 mg/kg /once daily, oral gavage).

Group 9 (12 rats): (The high-dose extract-treated group) received extract (200 mg/kg /once daily, oral gavage).

Group 10 (12 rats): received nano formulation (1 ml/rat/once, oral gavage).

Groups 6–10 (n = 12): each group was split into two subgroups: one sacrificed at 6 hours post-indomethacin for gastric ulcer assessment and the other at 12 hours for AKI. Doubling group size to n = 12 ensured n = 6 per endpoint (gastric vs. renal).

Gastrotoxicity and nephrotoxicity experiments were conducted following a modified version of the methodology proposed by Djahanguiri [32]. One hour following oral administration of either formulation or vehicle, groups 6–10 were subjected to an acute dosage of indomethacin (25 mg/kg, oral gavage) [33].

The animals were subjected to anesthesia using ketamine and euthanized 6 h after the indomethacin dose for gastroprotective assessment and 12 h after the indomethacin injection for renal assessment. The Kidney and stomach were extracted following experimental protocols. The gastric contents were obtained by dissecting the stomachs of the rats along the larger curvature. Each rat’s stomach and renal tissues were carefully excised and stored in a 10% formalin-saline solution for future histological analysis. The remaining part was cryopreserved using liquid nitrogen and stored at a temperature of −80℃ for subsequent tissue analysis. To minimize analytical bias, all biochemical assays were performed in a blinded manner.

#### 2.5.2. Gross mucosal damage assessment.

Each rat’s stomach was gently dried with two filter sheets after being washed with a cold saline solution. Then the stomach was stretched out carefully and taped to a corkboard. Then, to make it easier to evaluate any possible changes to the stomach’s mucosal integrity, digital pictures were taken. ImageJ software (Wayne Rasband, MD, USA) was used to analyze the photographs. The software was used to measure the ulcerations’ size, and then the ulceration ratio was computed. Each animal’s U.I was determined using the provided formula and the procedures outlined by Szabo and Hollander [34]:

U.I. = [the ulcerated region/the total stomach area] * 100. The percentage of inhibition against ulceration is computed using the formula: % ulcer inhibition = [(U.I. in ulcer group – U.I. in the test group) /U.I. in ulcer group] * 100.

#### 2.5.3. Microscopic examination of gastric ulcers.

Fixation of the stomach and kidney tissues for 72 hours at room temperature was done using a 10% neutral buffered formalin solution. Tissue samples were subjected to ethanol processing through a graded series of ethanol solutions, followed by clearing in two changes of xylene. Cleared tissues were then embedded in molten paraffin wax blocks. Five-micron-thick tissue sections were cut, and the portions underwent an H&E staining. Finally, stained sections were objectively evaluated by an experienced pathologist who was completely blinded to the experimental design and group allocations under light microscopy for histopathological changes.

#### 2.5.4. Measurement of gastric acidity.

A study on the acidity of gastric juice was conducted following Beiranvand et al.’s technique. To sum up, the stomach contents of all the rats were centrifuged for 10 minutes at 5000 rpm. After carefully collecting the resulting supernatant, 1 milliliter of the supernatant was mixed with the same volume of distilled water. After that, a pH meter (Fisherbrand, AB315 tabletop pH meter, Waltham, MA, USA) was used to determine the mixture’s pH [35,36].

#### 2.5.5. Biochemical analysis.

The renal functions were assessed through the quantification of serum creatinine using a Scr-determination kit (human diagnostic, Wiesbaden, Germany #10051) and the quantification of BUN levels using a BUN kit (human diagnostic, Wiesbaden, Germany #10505).

#### 2.5.6. Estimation of KIM-1 level.

The ELISA technique was employed to measure KIM-1 levels to identify AKI using Rat KIM kits (#CSB-E08808r, Cusabio in Houston, TX, USA).

#### 2.5.7. Evaluation of oxidative stress markers in gastric and renal tissue.

Using the colorimetric approach devised by Tappel and Zalkin [97], the measurement of lipid peroxidation byproducts, especially MDA, in the gastric or renal tissues was carried out as a tool to quantify oxidative stress. The findings were given in nmol per g of tissue. Using a commercially available kit, the SOD level was quantified to evaluate antioxidant indicators.

#### 2.5.8. Assessment of STAT-3 level by western blotting.

Using RIPA buffer (Abcam plc, Cambridge, MA) and standard protocols, tissue lysates were produced. Next, each sample’s protein content was ascertained using the Bradford Protein Assay Kit (Catalogue No. SK3041, Markham, Ontario, L3R 8T4, Canada). After separating 20 μg of protein from the samples using SDS-PAGE, the protein was electrotransferred onto polyvinylidene fluoride membranes. Bovine serum albumin was used to block the membrane for one hour. The primary rabbit anti-STAT3 (# 12640, Cell Signalling Technology, Danvers, MA, USA), rabbit anti-pY705 STAT3 (# 9145S, Cell Signalling Technology, Danvers, MA, USA), and β-actin (Santa Cruz Biotechnology, Santa Cruz, CA) were then added to the membranes and incubated for an additional night at 4 ◦C. Then, using goat anti-rabbit polyclonal immunoglobulin coupled with horseradish peroxidase (1:500) (Cell Signalling Technology Inc., MA, USA), an incubation period of four hours at room temperature was carried out.

Using ClarityTM Western ECL substrate (Bio-Rad, catalog number: 170–5060), an enhanced chemiluminescence (ECL) substrate reaction, the bands were identified. Image J was used to accomplish the quantification process.

#### 2.5.9. Assessment of p-NF-κB p65 level by ELISA.

Using the NF-κB-p65 assay kit (Elabscience, Biotech Inc., USA), the manufacturer’s suggested methods were followed to measure the amounts of NF-κB-p65 in the stomach and kidneys.

#### 2.5.10. Gene expression analysis.

***Total RNA extraction:*** A 50 mg sample was homogenized in 0.5 mL of TRIzol reagent (RNA Isolation Reagent, Invitrogen – ThermoFisher Products & Kits, Amresco, LLC-Solon, USA) using an ultrasonic homogenizer (Sonics-Vibracell, Sonics and Materials Inc., Newtown, Fairfield County, Connecticut, USA) [37]. The tissues were subjected to total RNA isolation using the method advised by the manufacturer. The evaluation of the concentrations of RNA purity and yield was done [38].

***Real-time–PCR:*** For the reverse transcription procedure, the Revert Aid H Minus First Strand cDNA Synthesis kit (#K1632, Thermo Scientific Fermentas, St. Leon-Ro, Germany) was utilised in compliance with the manufacturer’s instructions. In all cases, the same amount of total RNA was used. For the qRT-PCR assays in this paper, Thermo Scientific Fermentas, St. Leon-Ro, Germany, provided the Maxima SYBR Green reagent. The assortment of primers used for qRT-PCR is shown in (Table 1). The Applied Biosystems Step One qRT-PCR Detection System was used to perform the qRT-PCR analysis [39]. After measuring the levels of gene expression, the normalisation process was carried out with GAPDH serving as the reference gene. To assess the relative amounts of RNA, we employed the comparative Ct method. This was accomplished by applying the algorithm to determine the RQ value using the formula RQ = 2 ^(-ΔΔCt)^ [40].

**Table 1:** The real-time PCR primers.

*IL-1β*	Forward	5’-GTGATGAAAGACGGCACACC-3’
	Reverse	5’-TCCTGGGGAAGGCATTAGGA-3’
*GAPDH*	Forward	5’-CTCTCTGCTCCTCCCTGTTC-3’
	Reverse	5’-CGACATACTCAGCACCAGCA-3’
*TNF-α*	Forward	5’- CCTCTCTGCCATCAAGAGCC-3’
	Reverse	5’- GGCTGGGTAGAGAACGGATG-3’
*iNOS*	Forward	5′- CACCACCCTCCTTGTTCAAC −3′
	Reverse	5′- CAATCCACAACTCGCTCCAA −3′

#### 2.5.11. Statistical analysis.

The results are shown as mean ± SD. Multiple comparisons were performed using an ANOVA test, and further comparisons were performed using Tukey’s post hoc test. Software called GraphPad Prism 8 (GraphPad Software Inc., La Jolla, CA, USA) was used to perform the statistical analyses.

### 2.6. Bioinformatic study

#### 2.6.1. Plant-Cpd network.

Based on the metabolomic profiling of *Aerva javanica* using LC-HRESIMS, 27 dereplicated compounds, a plant–Cpd network was constructed.

#### 2.6.2. Cpd -Target network.

The databases PubChem(https://pubchem.ncbi.nlm.nih.gov/) [41], which was last accessed on November 3, 2023, and SwissTargetPrediction, which was last accessed on November 7, 2023, were employed to identify the primary Cpds in the *Aerva javanica* (http://www.swisstargetprediction.ch/) [42].

#### 2.6.3. Intersected genes determination.

FunRich software 3.1.3 was used to preserve the representation of the intersected genes, or genes having dual functions as nephroprotective and gastroprotective.

#### 2.6.4. Protein-Protein Interactions.

Using the online database STRING, the functional link between each of the selected targets (genes) was predicted in a network form and visualized (https://string-db.org/cgi/network?taskId=bgI0vTh97E1E&sessionId=bYFdOL7xOkeV) with Median confidence 0.4 [43], last accessed on (11-011-2023).

#### 2.6.5. Targets-gastric and nephrotic disorders network.

DisGeNet was used to gather the genes linked to nephrotic and stomach illnesses [44]. Every target was utilized in the DisGeNet database (under common names) to determine the gene connection with nephrotic and gastric illnesses. After performing a results filter, we eliminated uncommon illnesses and malignant conditions and narrowed our search results to just include gastric and nephrotic disorders.

#### 2.6.6. Network construction and visualization.

Utilizing the program Cytoscape version 3.9.0, networks related to plant-Cpd, Cpd-target, PPI, and target-nephrotic diseases, Cpd-gastric disorders, and Cpd-intersected genes were constructed [45]. In the graphical network, nodes stand in for plants, Cpd, targets, nephrotic diseases, and stomach disorders, while edges indicate related connections.

#### 2.6.7. Gene enrichment analysis.

Three databases were used to do the Gene Ontology and enrichment analysis for the genes with the dual activities (intersected genes); these databases included the WebGestalt database [46] (https://www.webgestalt.org/), ShinyGO database [47] 0.8 (http://bioinformatics.sdstate.edu/go80/), and SRPlot bioinformatics database [48] (https://www.bioinformatics.com.cn/basic_local_go_pathway_enrichment_analysis_122_en) to identify the Gene ontology in terms of biological process, cellular component, and molecular function. The enrichment analysis of the biological pathways was determined according to the KEGG database.

## 3. Results

### 3.1. Metabolomic profiling of the methanolic extract of *Aerva javanica* by LC-HR-ESI-MS

The chemical components in the methanolic extract of *Aerva javanica* that have the potential to be gastroprotective and nephroprotective are metabolically profiled using LC-HR-ESI-MS. 27 chemicals were tentatively identified in the crude extract by LC-HR-ESI-MS (S1 Table). The discovered compounds were enumerated and grouped according to their molecular weight (M.wt) in (S1 Table).

### 3.2. TEM characterization of the synthesized *Aerva javanica* SNPs

According to (Fig 1), the silver nanoparticles TEM investigation revealed spherical nanoparticles with a mean size of 20.59 nm.

**Fig 1:**
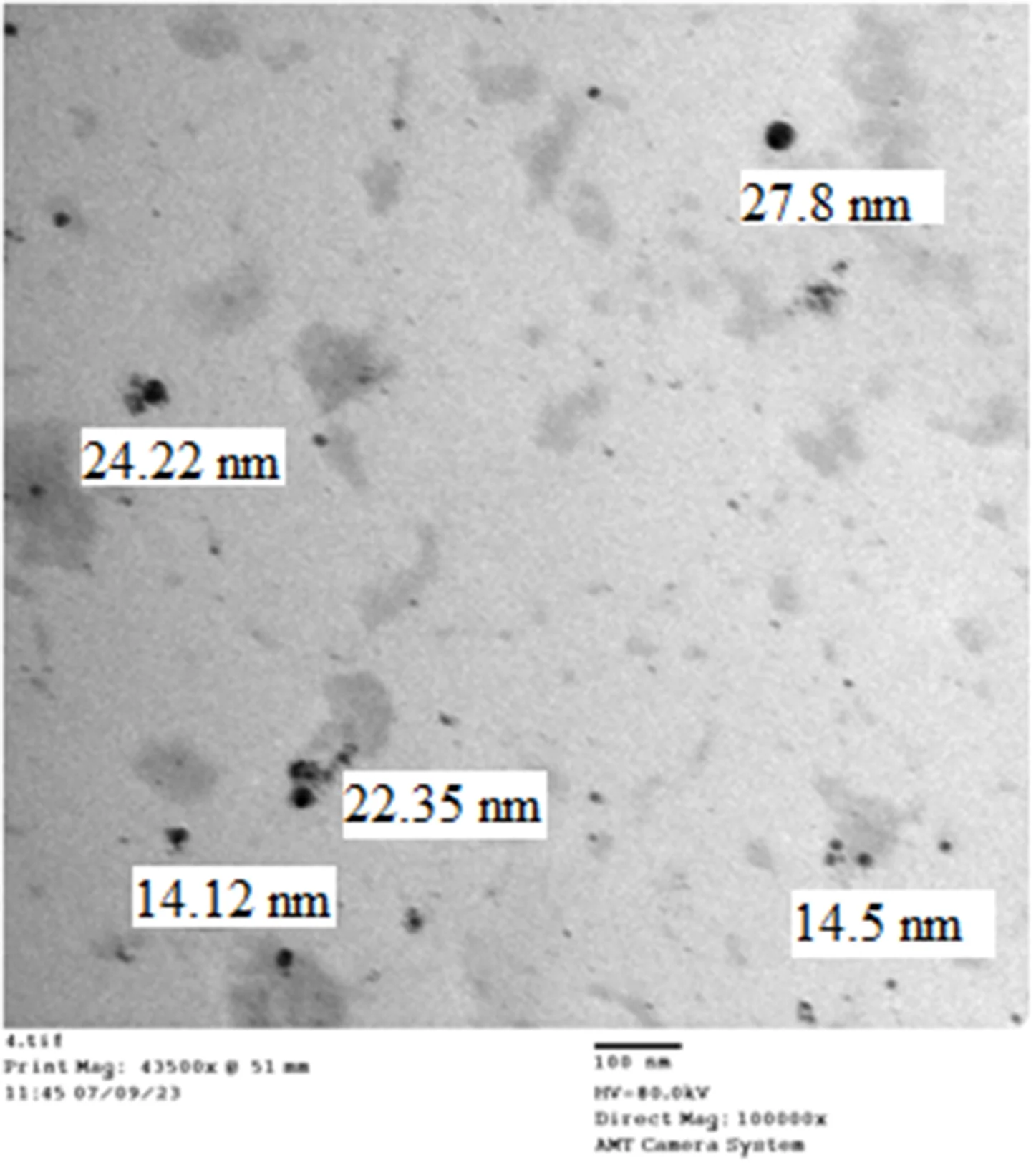
TEM examination of the synthesized SNP size and form in the methanolic extract of Aerva javanica.

### 3.3. UV-visible and FTIR characterization of the synthesized SNPs of *Aerva javanica* methanolic extract

When SNPs were initially synthesized, the surface plasmon resonance (SPR) phenomenon caused them to change color to a reddish-brown hue. By analyzing the reaction medium’s UV-Vis spectra in the 200–600 nm wavelength region, the generation of SNPs was tracked. For *Aerva javanica* synthesized SNPs, an absorbance band was seen at 415 nm (Fig 2A), which is consistent with those found in the literature and validates the formation of AgNPs [49].

**Fig 2:**
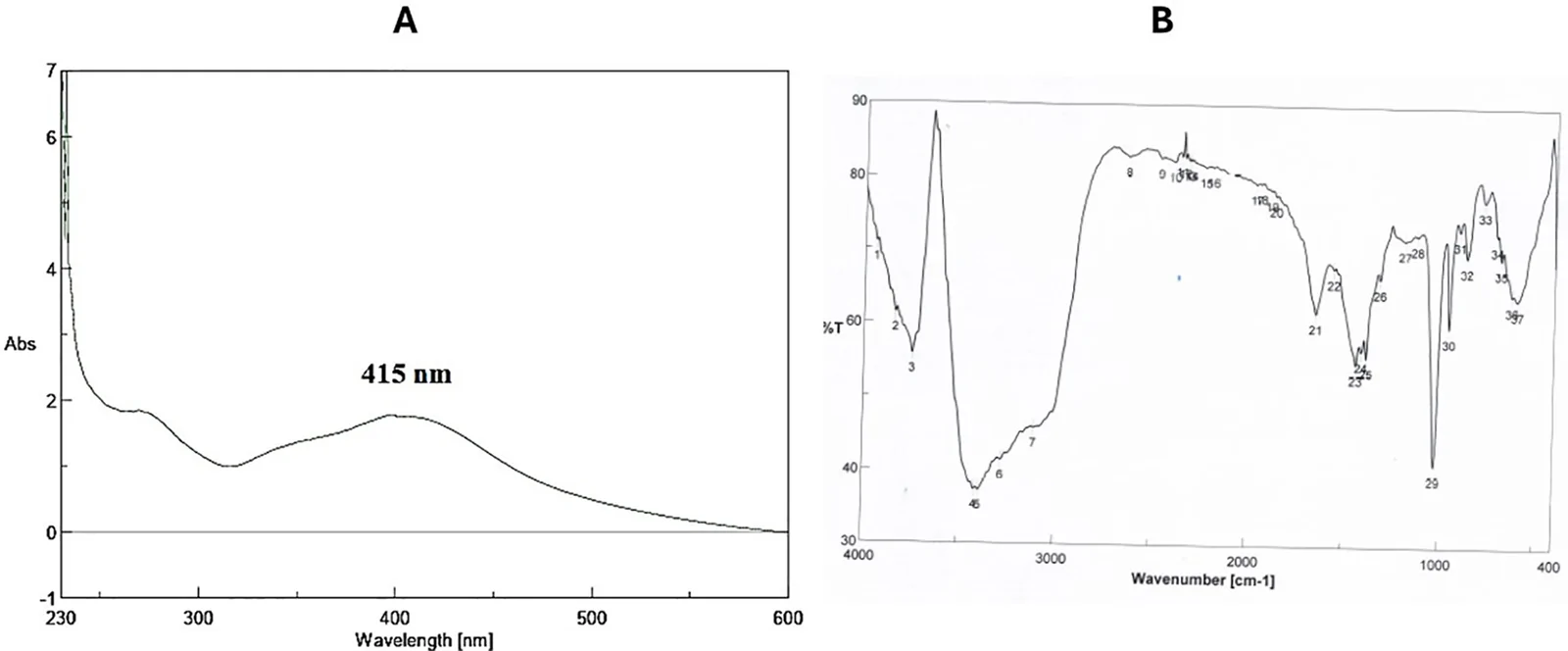
The biosynthesized SNPs of Aerva javanica were analyzed using UV-Vis, and the FTIR spectra of the SNPs of the methanolic extract were examined in (B).

These days, FTIR is an essential technique for understanding how functional groups interact with metal particles in biomolecules. It may be utilized to find certain biomolecules required for the efficient capping and stabilization of the metal nanoparticles, as well as to determine the surface makeup of the silver nanoparticles. The bio-reduction of Ag+ ions may have been caused by the many functional groups that are present.

Different significant peak locations at 3308.31, 3326.39, 2083.84, 1636.62, 1283.47, 544.01, and 458.19 cm^-1^ are displayed in the FTIR spectrum (Fig 2B). Stretching of the N-H group and –OH stretching in alcohols and phenolic compounds with strong hydrogen bonds were identified as the peaks in the 3,200–3,500 cm^-1^ range. The peak in the 2,083.12 cm^-1^ range is significant for the stretching of triple bonds. In the region of 1635.64 cm^-1^, the FTIR signal shows that the C = C bond is stretching. The peaks show the alkyl halides bond stretch in the range 505.35–428.20 cm^-1^. Terpenoids, flavonoids, glycosides, phenols, and tannins containing functional groups like ketone, aldehyde, carboxylic acid, and others are primarily responsible for the observed peaks [29,50]. The stability of the nanoparticles is increased by these groups’ presence. These metabolites stop the nanoparticles from pairing and aggregating. These biomolecules can be assumed to be in charge of capping and effectively stabilizing synthesized nanoparticles.

### 3.4. Nephroprotective and gastroprotective effect of *Aerva javanica* methanolic extract

#### 3.4.1. Effect of extract on ulcer index of indomethacin-treated rats.

Macroscopic examination of stomachs from control groups (1–5) revealed intact mucosa with no visible ulcers or inflammation. In addition, the mucosa had a normal, strong pink hue, unbroken folding patterns, and appropriate thickness indicative of mucosal integrity. In contrast, indomethacin-treated rats (group 6) exhibited pronounced hemorrhagic longitudinal irregular mucosal lesions of various sizes and depths. Pretreatment with low- or high-dose extract or nanoformulation yielded only moderate mucosal lesions and congestion. These results suggest that both the nanoformulation and the extract, when given at low and high dosages, had preventive benefits, with the nanoformulation having the highest protective potential. Following indomethacin administration, the ulcer index (UI) (11.2) was considerably higher than in the control group (p < 0.05). However, as compared to the group receiving indomethacin treatment, the administration of both low and high dosages of the extract led to a substantial decrease in UI (3.117 and 1.1, respectively) (p < 0.05). These results were comparable to famotidine’s effects (1.25). Moreover, the administration of the nanoformulation demonstrated a significant reduction in UI (0.17) in contrast to the group receiving indomethacin treatment. Notably, a statistically significant difference was seen between the groups that received nano treatment and the extract that was given at both dosages, as nanoformulation has a much higher protective capacity (Fig 3).

**Fig 3:**
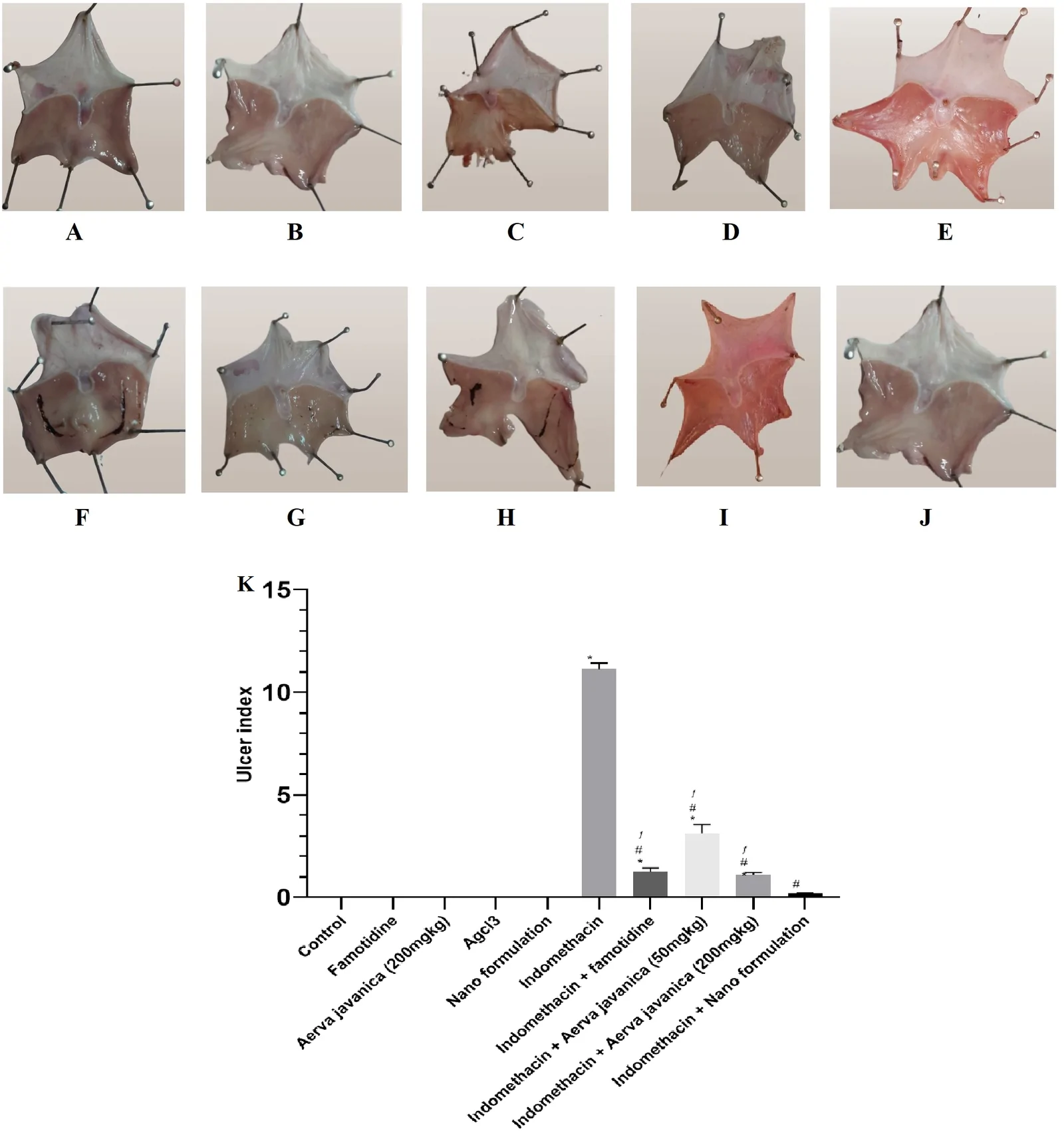
Illustrative photo macrographs of the gastric region in rats of different groups. (A) Control, (B) Reference drug (famotidine), (C) *A. javanica* extract 200 mg/kg, (D) Silver nitrate solution, (E) *A. javanica* nanoparticles, (F) Indomethacin, (G) Famotidine + Indomethacin (H) Indomethacin + *A. javanica* 50 mg/kg extract, (I) Indomethacin + *A. javanica* 200 mg/kg extract, (J) Indomethacin + *A. javanica* nanoparticles. (K) The impact of indomethacin, alone or in combination with famotidine, a tested extract (at doses of 50 and 200 mg/kg), as well as a Nano formulation, on the gastric UI in rats. The statistical analysis involved the use of one-way ANOVA followed by Tukey’s post hoc test to compare the means. The results are reported as mean ± SD. * Results were determined to be statistically significant compared to the control group, with a p-value less than 0.05. # results were determined to be significantly different from the group treated with indomethacin alone (ulcer), with a p-value less than 0.05. ƒ Results were found to be substantially different from the group treated with *A. javanica* nanoparticles. with a p-value less than 0.05.

#### 3.4.2. Histopathological examination.

**Histopathological analysis of gastric tissue:** Groups from 1–5: Following hematoxylin and eosin (H&E) staining, histopathological examination showed healthy gastric mucosa without surface epithelial ulceration or shedding, normally arranged gastric foveolar glands without necrosis, and healthy noncongested nonedematous lamina and submucosa, as shown in (Fig 4a-e). Group 6: Histopathological examination of gastric tissue showed ulcerated surface epithelium covered with necrotic tissue clumps, foci of glandular necrosis with distorted architecture, with congested and edematous lamina and submucosa, as shown in (Fig 4f). Group 7: The examined gastric tissue showed slightly unhealthy surface mucosa with a focal area of epithelial desquamation. Foveolar glandular architecture is preserved with mild submucosal congestion (Fig 4g). Group 8: The section examined showed healthy surface mucosa without ulceration or necrosis, with a small focal area of epithelial debris. Foveolar glandular architecture is preserved and regenerated, and no submucosal congestion or edema, as shown in (Fig 4h). Group 9: The histopathological section showed healthy surface mucosa without ulceration or necrosis. Foveolar glandular architecture is preserved and regenerated. No submucosal congestion or edema (Fig 4i). Group 10: Histopathological examination of gastric tissue showed healthy surface mucosa without ulceration or necrosis, covered with eosinophilic secretion. Foveolar glandular architecture is preserved and regenerated, and no submucosal congestion or edema except at one capillary, as shown in (Fig 4i).

**Fig 4:**
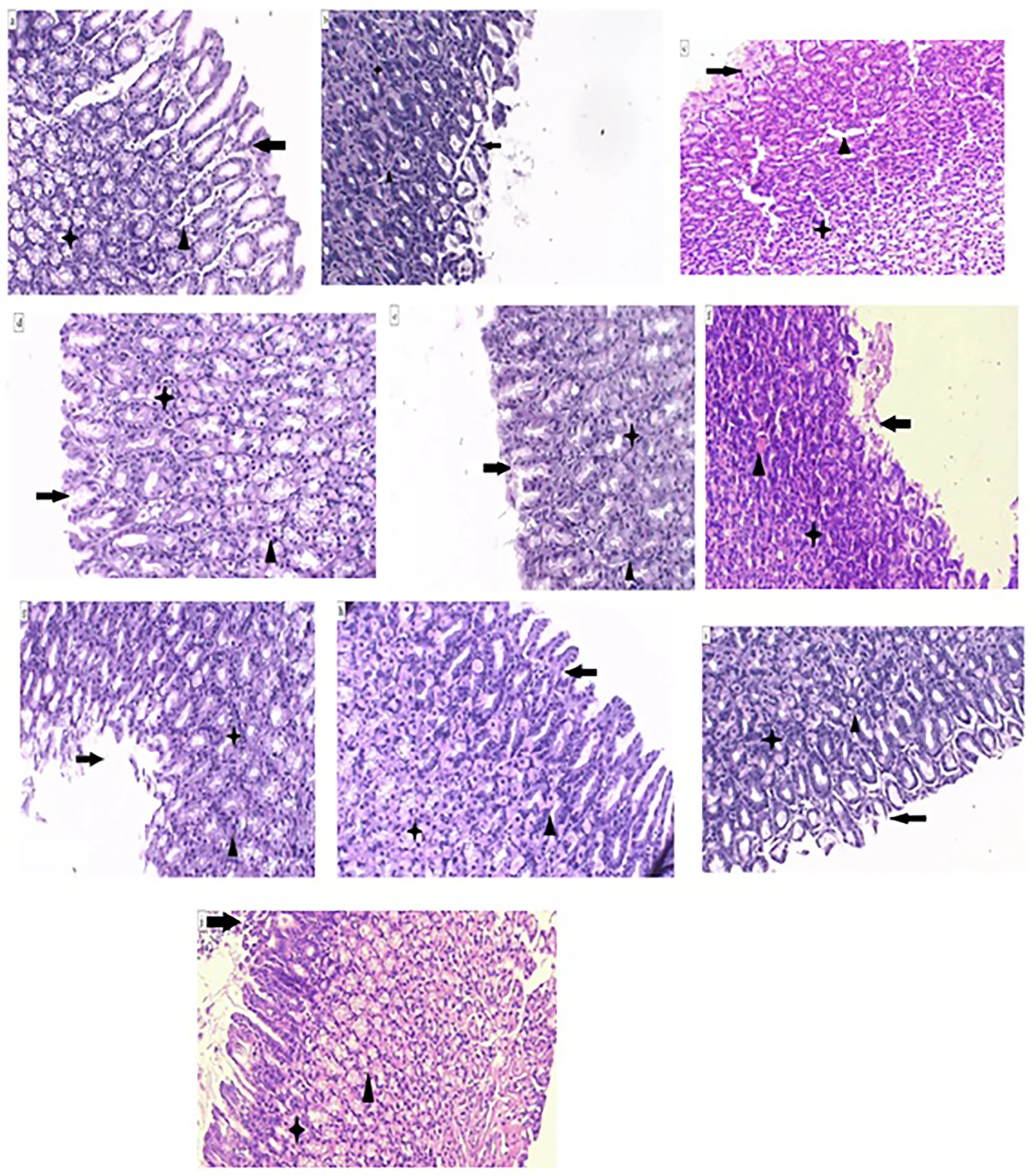
Histopathological examination of gastric tissues: (a-e): The section examined showed healthy gastric mucosa without surface epithelial ulceration or shedding (arrow), normally arranged gastric foveolar glands without necrosis (arrowhead), healthy non-congested nonedematous lamina and submucosa (asterix). (f) The section examined showed ulcerated surface epithelium covered with necrotic tissue clumps (arrow), foci of glandular necrosis with distorted architecture (arrowhead), and congested and edematous lamina and submucosa (asterix). (g) The section examined showed slightly unhealthy surface mucosa with a focal area of epithelial desquamation (arrow). Foveolar glandular architecture is preserved (arrowhead) with mild submucosal congestion (asterix). (h) The section examined showed healthy surface mucosa without ulceration or necrosis, with a small focal area of epithelial debris (arrow). Foveolar glandular architecture is preserved and regenerated (arrowhead). No submucosal congestion or edema (asterix). (i): The section examined showed healthy surface mucosa without ulceration or necrosis (arrow). Foveolar glandular architecture is preserved and regenerated (arrowhead). No submucosal congestion or edema (asterix). (j): The section examined showed healthy surface mucosa without ulceration or necrosis covered with eosinophilic secretion (arrow). Foveolar glandular architecture is preserved and regenerated (arrowhead). No submucosal congestion or edema except at one capillary (asterix). All Figs (200x).

**Histopathological examination of renal tissues:** Groups from 1 & 3–5: Histopathological examination of renal tissue at 12 hours showed normal tubular architecture without tubular cellular edema or intratubular eosinophilic casts and interstitial tissue without hemorrhage, inflammatory infiltrates or necrosis, as shown in (Fig 5a-d respectively). Group 6: Histopathological examination of renal tissue at 12 hours showed the normal histological appearance of glomeruli, mild tubular edema with scattered intratubular eosinophilic casts, and interstitial tissues with mild hemorrhage, and few inflammatory infiltrates. Group 8: Examined tissue at 12 hours, showed renal tissue with normal histological appearance of glomeruli with normal tubular lining without edema and intratubular eosinophilic casts and interstitial tissues without hemorrhage or inflammatory infiltrates. Group 9: Histopathological examination of renal tissue at 12 hours showed renal tissue with normal histological appearance of glomeruli, normal tubular architecture without tubular cellular edema or intratubular eosinophilic casts, and interstitial tissue without hemorrhage, inflammatory infiltrates, or necrosis, as shown in (Fig 5g). Group 10: Examined renal tissue at 12, showed the normal histological appearance of glomeruli, with normal tubular architecture without tubular cellular edema or intratubular eosinophilic casts, and interstitial tissue without hemorrhage, inflammatory infiltrates, or necrosis (Fig 5h).

**Fig 5:**
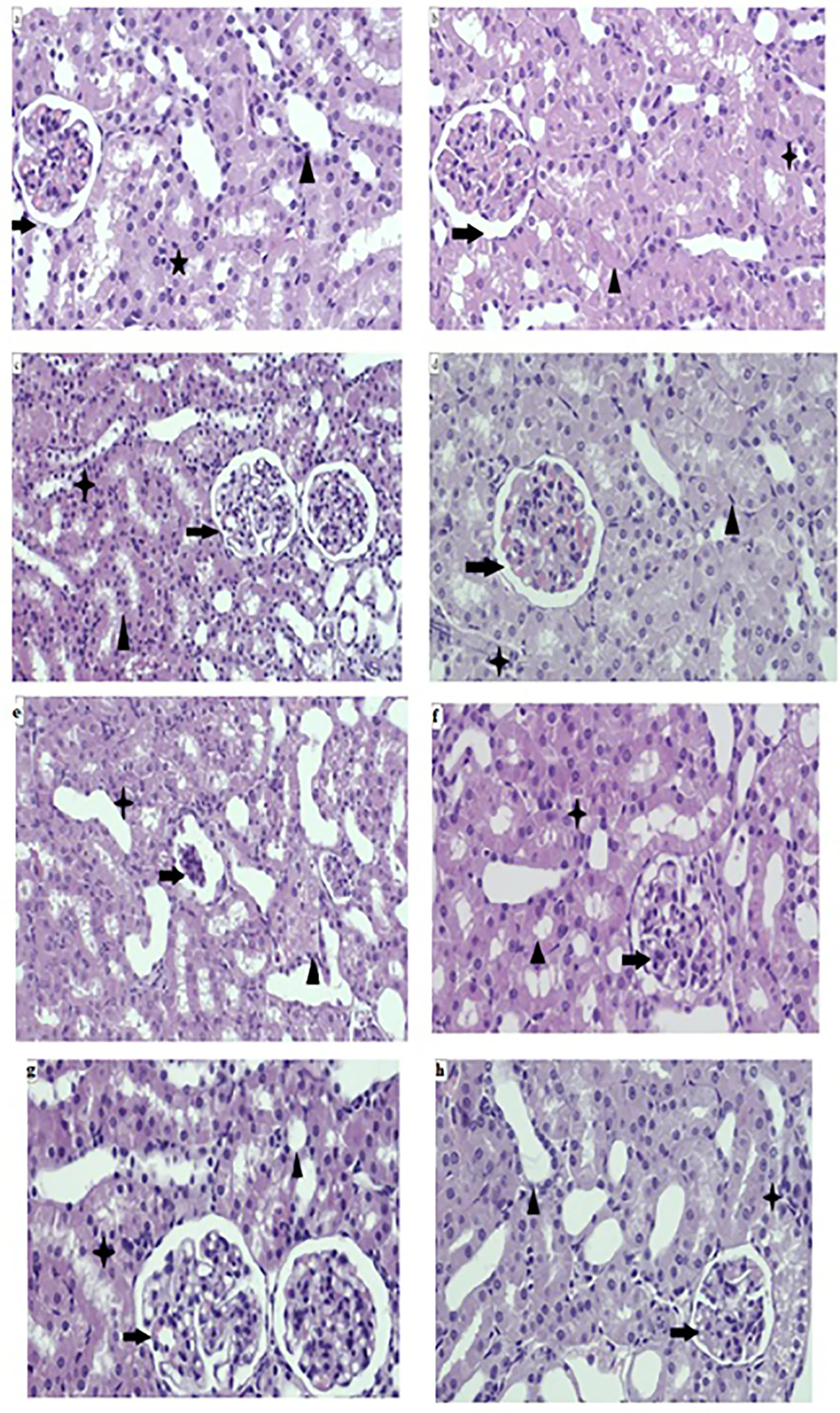
Histopathological examination of renal tissues: (a-d) Sections examined showed renal tissue with normal histological appearance of glomeruli (arrow), normal tubular architecture without tubular cellular edema or intratubular eosinophilic casts (arrowhead), and interstitial tissue without hemorrhage, inflammatory infiltrates, or necrosis. (e) Sections examined at 12 hours showed renal tissue with glomerular damage (arrow), mild tubular edema with scattered intratubular eosinophilic casts (arrowhead), and interstitial tissues with mild hemorrhage, few inflammatory infiltrates (asterix). (f) Sections examined at 12 hours showed renal tissue with normal histological appearance of glomeruli (arrow), normal tubular lining without edema and intratubular eosinophilic casts (arrowhead), and interstitial tissues without hemorrhage or inflammatory infiltrates (asterix). (g) Sections examined showed renal tissue with normal histological appearance of glomeruli (arrow), normal tubular architecture without tubular cellular edema or intratubular eosinophilic casts (arrowhead), and interstitial tissue without hemorrhage, inflammatory infiltrates, or necrosis (asterix) at 12 hours. (h)The sections examined showed renal tissue with normal histological appearance of glomeruli (arrow), normal tubular architecture without tubular cellular edema or intratubular eosinophilic casts (arrowhead), and interstitial tissue without hemorrhage, inflammatory infiltrates, or necrosis (asterix). All Figs (200x).

#### 3.4.3. Effect of extract on gastric pH.

One particularly interesting result of the study was the clear increase in stomach acidity, as demonstrated by a notably lower gastric pH in rats treated with indomethacin than in control rats (Fig 6, p < 0.05). However, in comparison to the indomethacin group, the administration of either famotidine or extract (at low or high doses) successfully reduced the levels of stomach acidity (4.1, 3.7, and 4.07, respectively) (p < 0.05). Moreover, compared to the group receiving indomethacin treatment, the administration of the nanoformulation led to a significant reduction in stomach acidity (4.25). Notably, a statistically significant difference was seen between the groups that received nano treatment and the extract that was given at both dosages, with nanoformulation having the higher pH value (Fig 6).

**Fig 6:**
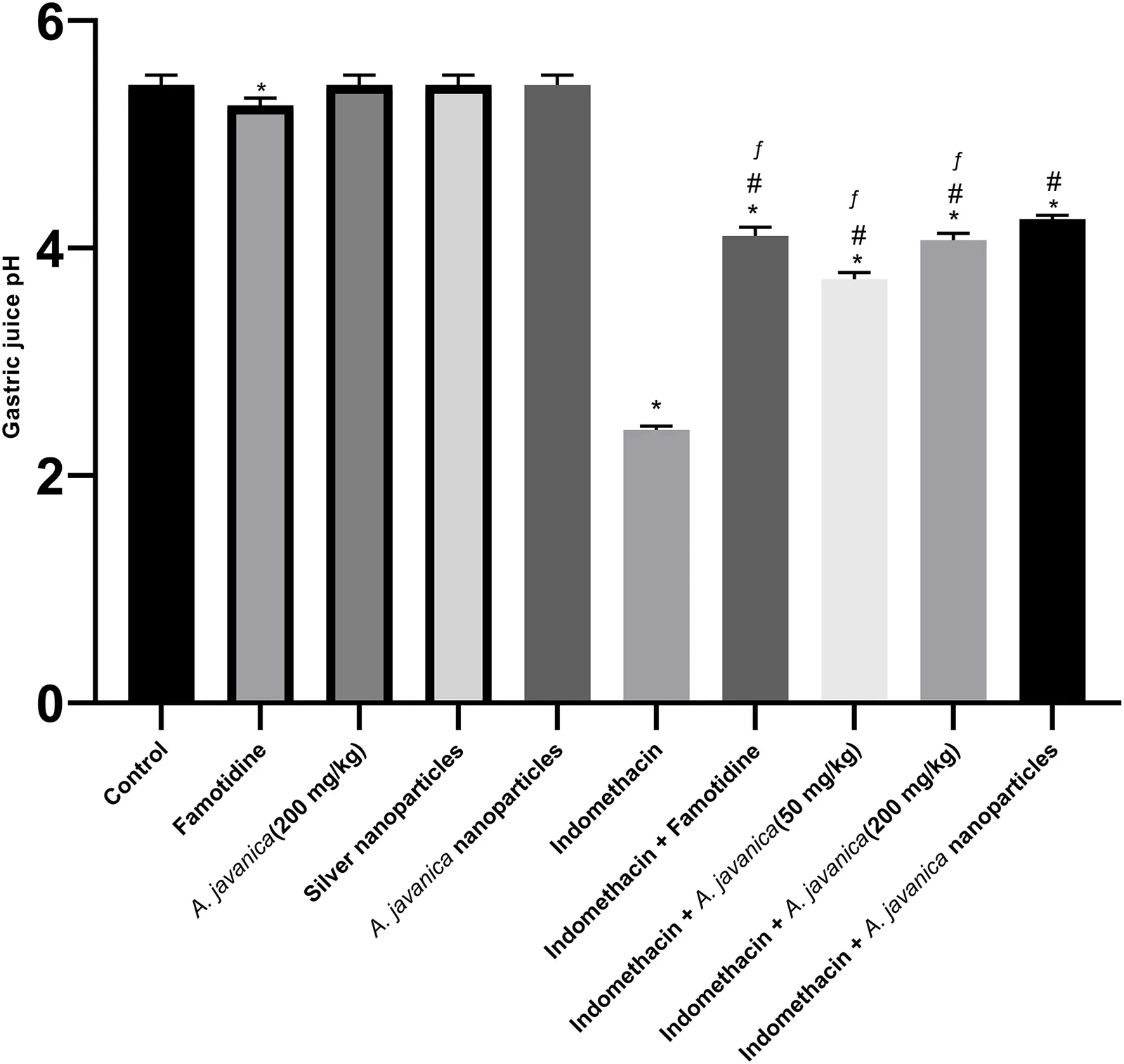
This study looks at how indomethacin affects the pH of rats’ stomachs when given either alone or in combination with famotidine, a tested extract (50 or 200 mg/kg), and *A. javanica* nanoparticles. To find any significant differences, a one-way ANOVA was used in the statistical analysis, and Tukey’s post hoc test was then performed. The findings are given as mean ± SD. Statistical analysis revealed that the * findings differed significantly from the control group (p-value < 0.05). # findings showed a significant difference (p-value < 0.05) from the group receiving indomethacin alone (ulcer) treatment. ƒ with a p-value less than 0.05, it was shown that the findings were statistically different from the group that received *A. javanica* nanoparticle treatment.

#### 3.4.4. The determination of the markers of renal toxicity.

The observed data regarding the indicators suggestive of renal toxicity are shown in (Table 2). Concerning the biochemical evaluations, blood urea nitrogen (BUN) and serum creatinine concentrations were measured. When compared to the control group, the BUN and serum creatinine levels in the indomethacin-treated group were significantly higher, coming in at 48.9 and 6.2, respectively. Meanwhile, compared to the rats injected with indomethacin, the experimental rats’ BUN (28.74 and 25.12) and serum creatinine (4.2 and 3.12) concentrations significantly decreased after the extract (50 mg/kg or 200 mg/kg) was administered. Additionally, the rats that received the nanoformulation injection exhibited a more significant decrease in the evaluated indicators (BUN: 20.27, serum creatinine: 2.54) compared to the group that was administered indomethacin. Furthermore, the nanoformulation has the highest protective activity compared to the extract at either the low or high dose (Table 2).

**Table 2:** The effect of the tested extract (50, 200 mg/kg) or nanoformulation on the indicators of nephrotoxicity in an experimental rat model of acute renal toxicity caused by indomethacin.

Treated groups	BUN (mg/dl)	Serum creatinine (mg/dl)
Control	17.74	2.1
Extract 200 mg	15.46	2.05
AgNO_3_	16.88	1.98
Nano formulation	16.97	2.15
Indomethacin	48.9*	6.2*
Indomethacin + Extract 50 mg	28.74^*, #, ƒ^	4.2^*, #, ƒ^
Indomethacin + Extract 200 mg	25.12^*, #, ƒ^	3.12^*, #, ƒ^
Indomethacin + Nano particles	20.27^*, #^	2.54^*, #^

Assessments of renal biomarkers were carried out after the trial was finished. The average of the data is shown. ANOVA was used for the statistical analyses, and the Tukey test was used as a post hoc analysis. * Results were determined to be statistically significant compared to the control group, with a p-value less than 0.05. # results were determined to be significantly different from the group treated with indomethacin alone (ulcer), with a p-value less than 0.05. ƒ Results were found to be substantially different from the group treated with *A. javanica* nanoparticles. with a p-value less than 0.05.

#### 3.4.5. Effect of extract on kidney injury molecule-1 (KIM-1) level.

KIM-1 has been observed to be prominently upregulated in renal tissues of animal models in response to acute kidney injury (AKI). In our investigation, within the animals subjected to kidney injury via the administration of indomethacin, the levels of KIM-1 exhibited a statistically significant elevation of 3.68 ng/mL in contrast to the control group (P  <  0.05) (Fig 7). This observation indicates the successful induction of AKI. The extract-treated groups at both doses, or nano formulation-treated groups, exhibited notable enhancements in comparison to the indomethacin group. Nevertheless, the most remarkable discovery seems to be the nanoformulation. At the dose of 50 mg/kg, it was observed that the extract resulted in a decrease in KIM-1 levels (1.9 ng/mL) relative to the group treated with indomethacin (P  <  0.05). At a dosage of 200 mg/kg, the group that received the extract exhibited notably reduced levels of KIM-1 (1.2 ng/mL) in comparison to the group that received indomethacin (P  <  0.05). In comparison to the indomethacin group, the utilization of the nanoformulation seemed to result in a convergence of KIM-1 values (0.87 ng/mL) towards those observed in the control group. Also, nanoparticle formulation showed greater protective efficacy compared to the crude extract (Fig 7).

**Fig 7:**
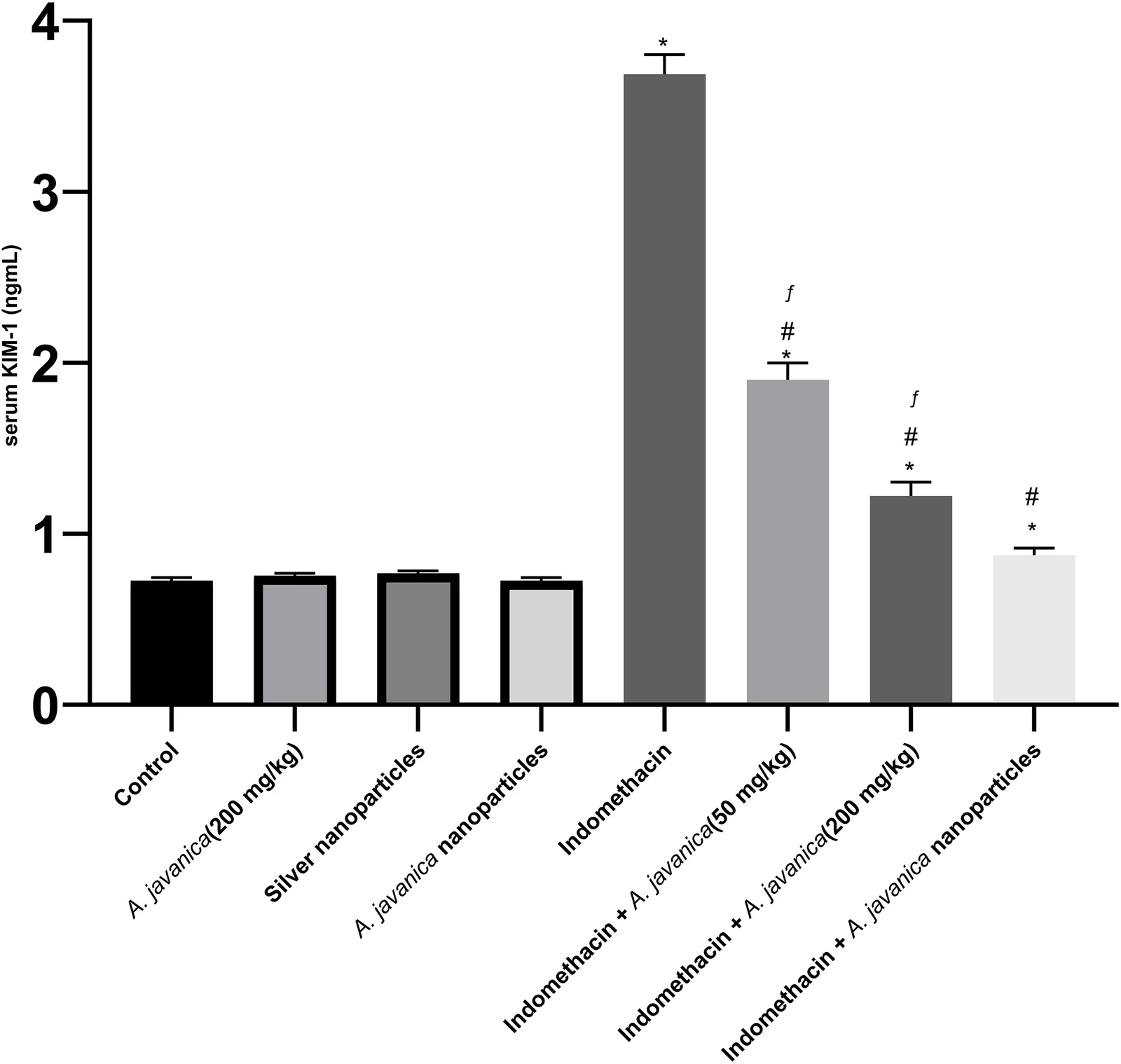
KIM-1 serum levels. Bars display mean ± SD. * ANOVA and Tukey tests were performed as post hoc analyses. * Results were determined to be statistically significant compared to the control group, with a p-value less than 0.05. # results were determined to be significantly different from the group treated with indomethacin alone (ulcer), with a p-value less than 0.05. ƒ Results were found to be substantially different from the group treated with *A. javanica* nanoparticles. with a p-value less than 0.05.

#### 3.4.6. The identification of oxidative stress Markers.

The effects of the extract, nano formulation, and/or indomethacin on the concentrations of MDA and SOD in the stomach and kidney tissues are shown in (Fig 8). When compared to the control group, the levels of SOD in the renal and gastric tissues were significantly reduced by indomethacin, coming down to 2.27 U/gm in the stomach and 2.047 U/gm in the kidney. As opposed to the group that received indomethacin, rats that received extract therapy at a dose of 50 mg/kg showed a significant increase in SOD levels in the kidney and stomach. The stomach’s SOD levels were significantly elevated after therapy at a dose of 200 mg/kg; they measured 4.5 U/gm tissue. Comparing the kidney to the group receiving indomethacin, the latter showed a SOD level of 4.12 U/gm tissue. Furthermore, compared to the group receiving indomethacin, the administration of nanoformulation therapy was associated with an elevation of SOD levels in the stomach (5.3 U/gm tissue) and the kidney (5.015 U/gm tissue). Additionally, it was noted that the rats given indomethacin had significantly higher levels of MDA in their stomach (6.6 nmol/gm tissue) and kidney (8.65 nmol/gm tissue) when compared to the control group. The administration of a modest dose of the extract led to a considerable drop in their concentration in the stomach to 3.85 nmol/gm tissue and in the kidney to 6.35 nmol/gm tissue, respectively, in contrast to the indomethacin group. Moreover, the therapy administered at a 200 mg/kg dosage led to a significant decrease in the stomach’s MDA content, which was measured at 2.9 nmol/gm tissue. Similarly, MDA levels in the kidney decreased as a result of the therapy (4.65 nmol/gm tissue. Compared to the group that got indomethacin, it is noteworthy that the use of nanoformulation therapy decreased the concentration of MDA in the kidney (measured at 3.8 nmol/gm of tissue) and stomach (2.49 nmol/gm of tissue). Moreover, nanoparticle formulation showed greater protective efficacy against oxidative stress compared to the crude extract.

**Fig 8:**
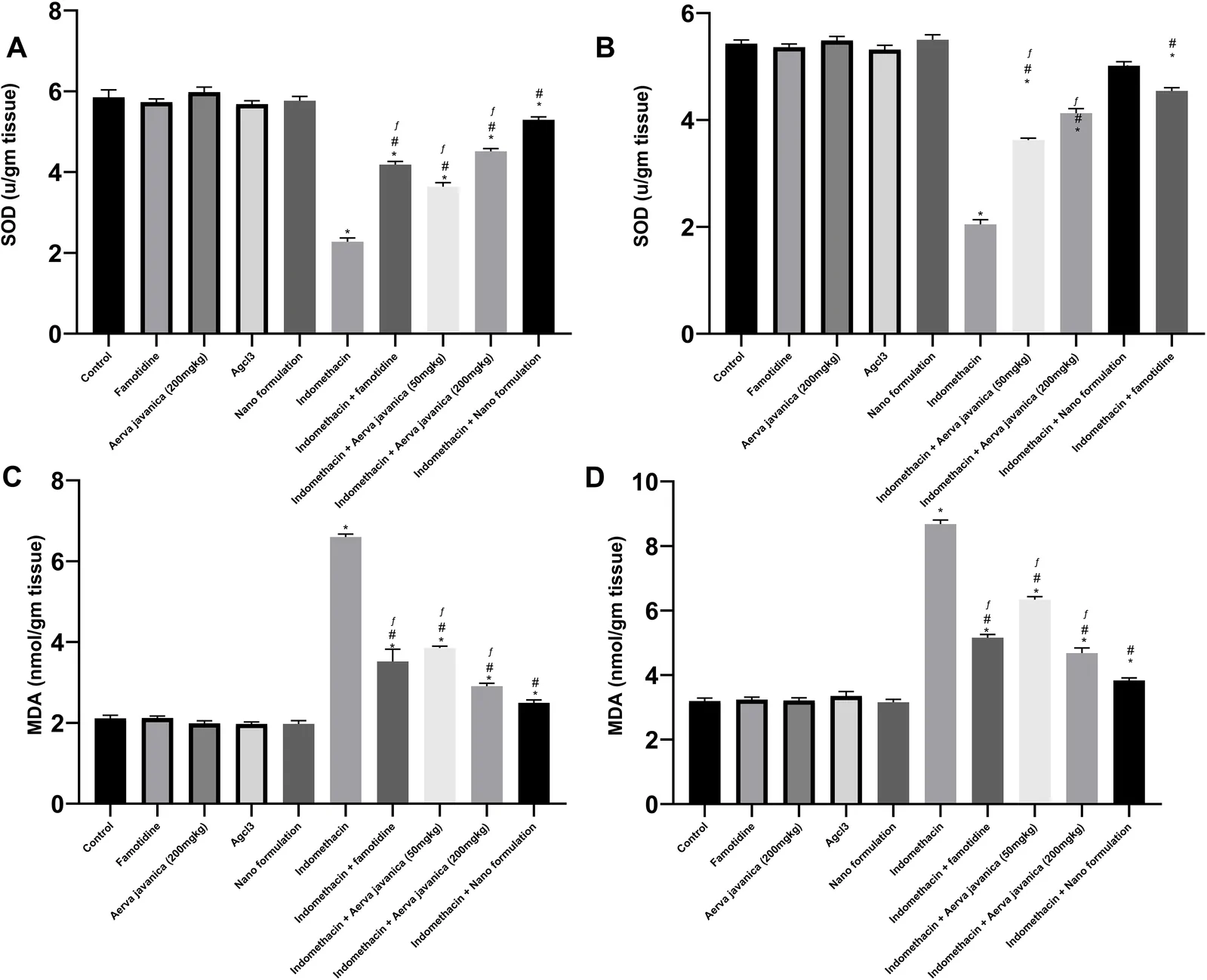
The effects of indomethacin on (A) gastric SOD, (B) renal SOD, (C) gastric MDA, and (D) renal MDA, either alone or in conjunction with oral administration of famotidine or *A. javanica* extract. ANOVA was used for the statistical analysis, and Tukey’s post hoc test was then performed. The mean ± SD is used to show the findings. * Results were determined to be statistically significant compared to the control group, with a p-value less than 0.05. # results were determined to be significantly different from the group treated with indomethacin alone (ulcer), with a p-value less than 0.05. ƒ Results were found to be substantially different from the group treated with *A. javanica* nanoparticles. with a p-value less than 0.05.

#### 3.4.7. Impact of the extract on the relative expression of genes.

The treatment of indomethacin resulted in a notable inflammatory response in the renal and gastric tissue, as seen by the considerable increases in TNF-α and IL-Iβ levels in the stomach and kidney relative to the control group (p < 0.05). Notably, the stomach’s elevated expression of TNF-α (5.2-fold and 4.1-fold) and IL-Iβ (3.86-fold and 2.45-fold) was significantly reduced (p < 0.05) when either low or high dosages of the extract were administered. Comparing this decrease to the indomethacin group revealed a dose-dependent pattern. Moreover, the nanoformulation showed an unexpected result: compared to the indomethacin group (p < 0.05), the stomach’s levels of inflammatory markers, TNF-α (3.5-fold) and IL-Iβ (1.72-fold), were considerably lower (Fig 9). Gene expression patterns in renal tissues were evaluated, and the results showed that the extract, at both low and high doses, significantly reduced TNF-α levels by 4.6 and 3 fold, respectively, and lowered IL-Iβ levels by 4.9 and 3.6 fold, respectively, compared to the indomethacin group (p < 0.05). The administration of the nanoformulation was shown to have a significant effect, as evidenced by a two-fold and three-fold decrease in the levels of inflammatory markers (TNF-α and IL-Iβ) in comparison to the group that received indomethacin treatment. It was found that nanoformulation has the highest protective activity compared to the extract at either the low or high dose (Fig 9).

**Fig 9:**
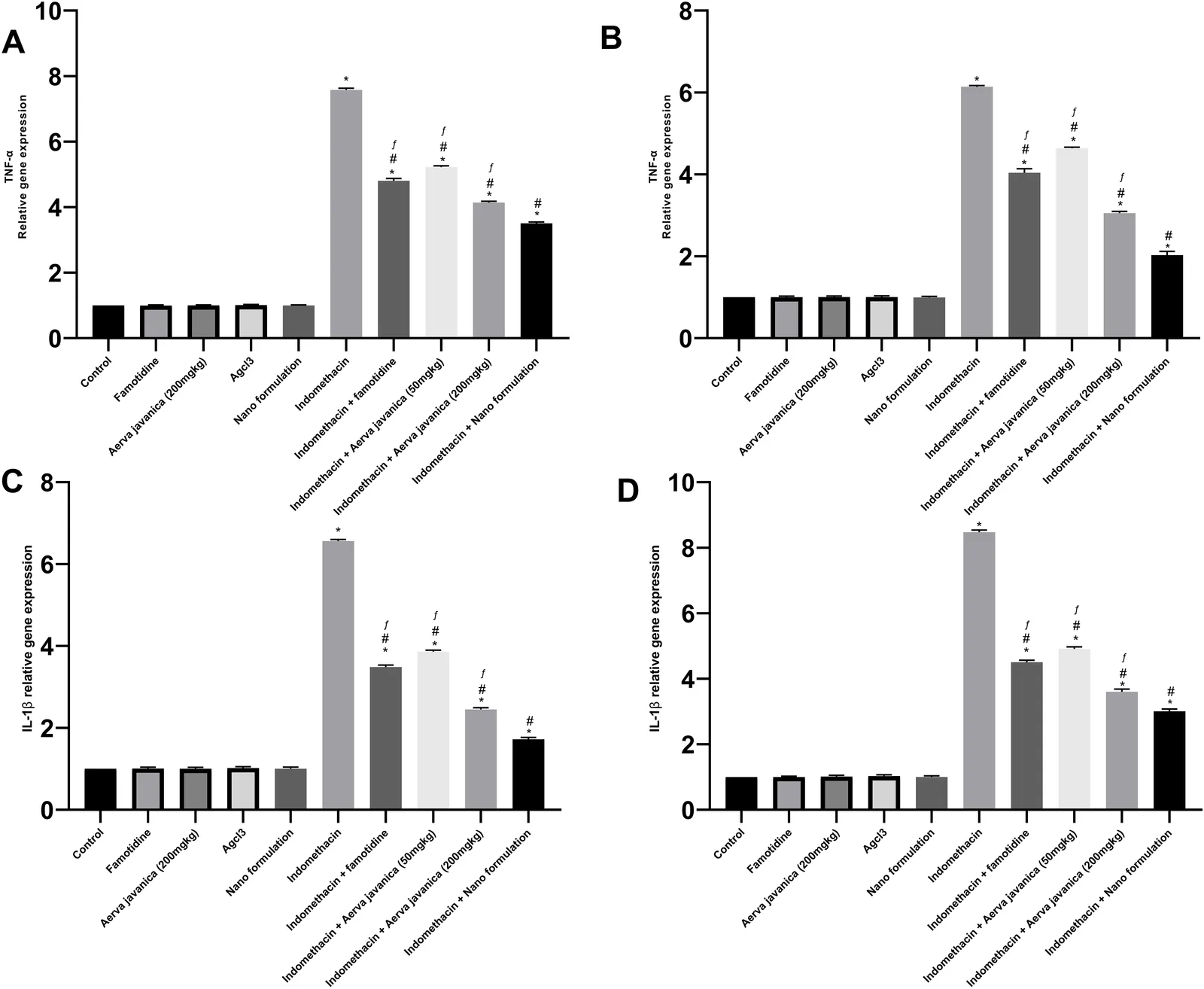
Relative expression of gastric and renal pro-inflammatory cytokine (A) gastric TNF-α, (B) renal TNF-α, (C) gastric IL-Iβ and (D) renal IL-Iβ in all study groups was detected by qRT-PCR. After normalizing to GAPDH, the data were presented as the ratio of the experimental group to the control group. The bars represent mean ± SD. ANOVA test, followed by Tukey’s post hoc test, was utilized to ascertain the significant distinction among the study groups. * The experimental group showed a statistically significant difference compared to the control group with p < 0.05. # The experimental group showed a statistically significant difference compared to the ulcer group with p < 0.05. ƒ: the observed disparities in the experimental group were statistically significant when compared to the group treated with *A. javanica* nanoparticles, with p < 0.05.

#### 3.4.8. Effect of extract on p-NF-κB p65.

Subsequently, we assessed the concentrations of NF-κB, an essential component of the traditional inflammatory cascade, in the stomach and kidney tissues. In comparison to the control group, Fig 10 shows a considerable increase in the protein expression levels of p-NF-κB p65 in the stomach (176 pg/mg protein) and kidney (165.2 pg/mg protein) following the injection of indomethacin. When the extract was administered at low or high dosages, the levels of p-NF-κB p65 protein expression in the stomach (152.7 and 140 pg/mg protein) and kidney (139.3 and 129.7 pg/mg protein) were significantly suppressed. There was a noticeable reliance of this inhibition on the dose that was given. As shown in (Fig 10), the use of nanoformulation also resulted in a much more decrease in the elevated amounts of renal (115.2 pg/mg protein) and gastric (131.5 pg/mg protein) p-NF-κB p65 proteins as compared to the indomethacin group and the groups that received extract at both doses (p < 0.05).

**Fig 10:**
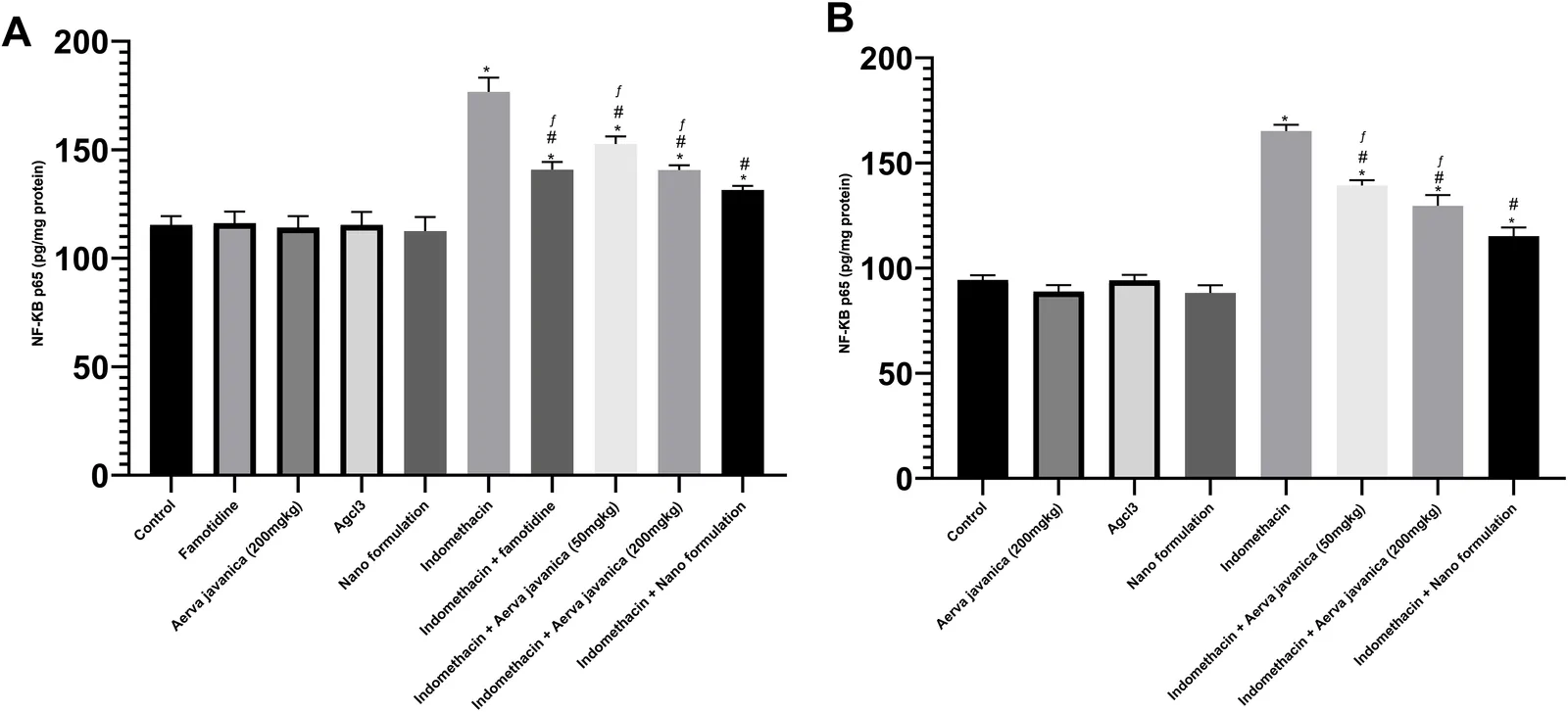
ELISA was used to determine the p-NF-κB p65 concentration in the stomach (A) and renal tissues (B) of each research group. The bars show the mean ± SD. To find any significant differences, a one-way ANOVA test was used to compare the study groups, and Tukey’s post hoc test was then performed. * Results were determined to be statistically significant compared to the control group, with a p-value less than 0.05. # results were determined to be significantly different from the group treated with indomethacin alone (ulcer), with a p-value less than 0.05. ƒ Results were found to be substantially different from the group treated with *A. javanica* nanoparticles. with a p-value less than 0.05.

#### 3.4.9. Effect of extract on iNOS level.

The gastric and renal tissues from a rat model that demonstrated indomethacin-induced gastrointestinal and renal damage had their levels of iNOS evaluated. Compared to the control group, there was a discernible presence of iNOS in the renal and gastric tissues in the indomethacin-treated group. The expression of iNOS in the stomach and kidneys was significantly reduced in rats given either a high or low dosage of the extract. Comparing the decline to the group that had indomethacin therapy, it was shown to be statistically significant (p < 0.05). When the nanoformulation was administered, the high levels of iNOS were significantly reduced (p < 0.05) in comparison to the group that received indomethacin treatment and extact groups at both doses (Fig 11).

**Fig 11:**
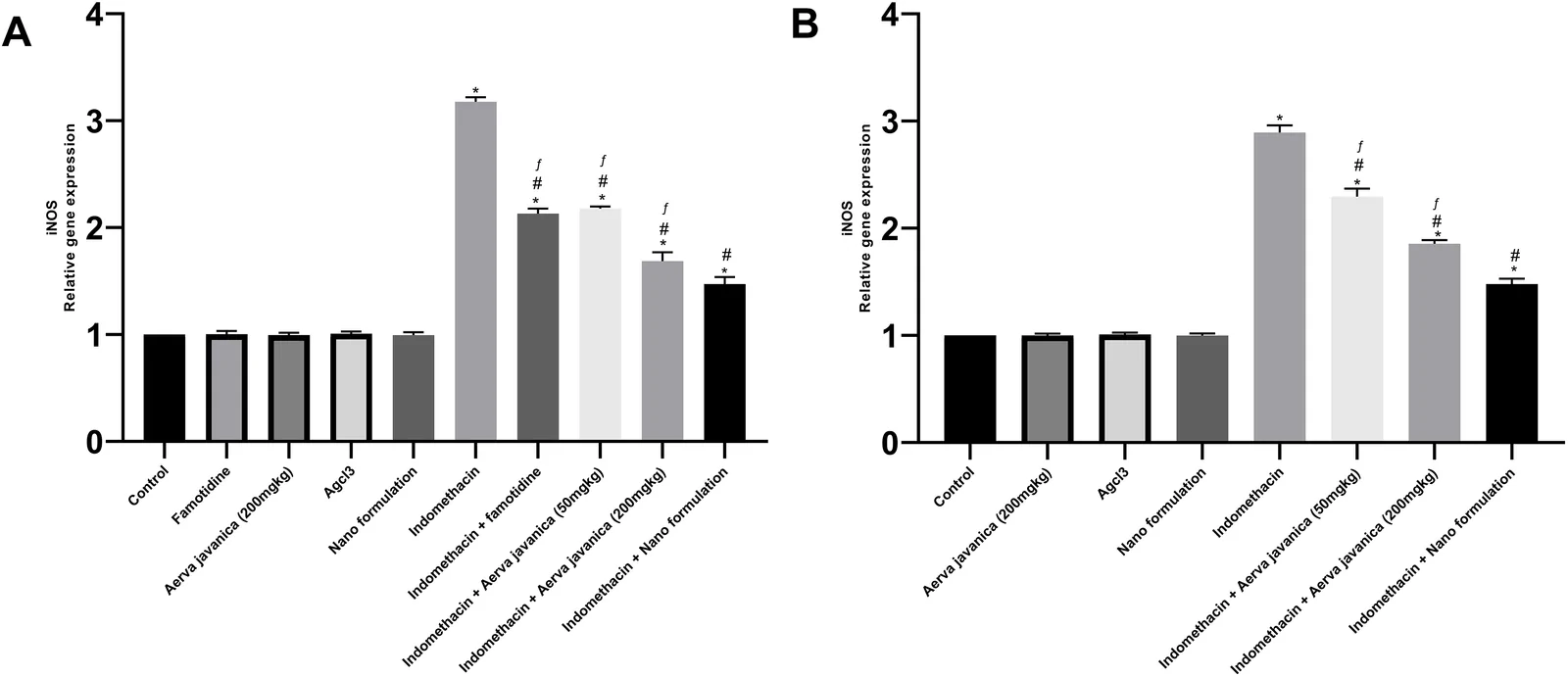
Using qRT-PCR, the amount of iNOS in the renal and stomach tissues in each research group was determined. The results were shown as the ratio of the experimental group to the control group after being normalized to GAPDH. The mean ± SD is shown by the bars. The ANOVA test was utilized to evaluate any significant differences between the research groups, and Tukey’s post hoc test was then performed. * Designate a p-value of less than 0.05 as a significant deviation from the control group. # and ƒ, respectively, denote significant differences between the groups receiving indomethacin alone (p < 0.05) and *A. javanica* nanoparticles (p < 0.05).

#### 3.4.10. Impact of the extract on the level of STAT-3.

The canonical STAT-3 expression levels in the stomach and kidney tissues were then investigated. The treatment of indomethacin led to a significant rise in the expression levels of STAT-3 protein in both the gastric and renal tissues, as shown in (Fig 12), which is a fold increase over the control group. Moreover, the extract at 50 mg/kg was found to have a significant inhibitory effect on STAT-3 protein expression levels in gastric and renal tissues as compared to those produced by indomethacin. Similarly, as compared to the group receiving indomethacin treatment, the extract administration at a dose of 200 mg/kg significantly decreased the abnormally elevated levels of renal and gastric STAT-3 proteins (p < 0.05). The elevated levels of renal and gastric STAT-3 proteins were significantly lower in the nanoformulation group than in the indomethacin group (p < 0.05). Also it was found that nanoformulation has the highest protective activity compared to the extract at either the low or high dose.The observed data are shown visually in (Fig 12).

**Fig 12:**
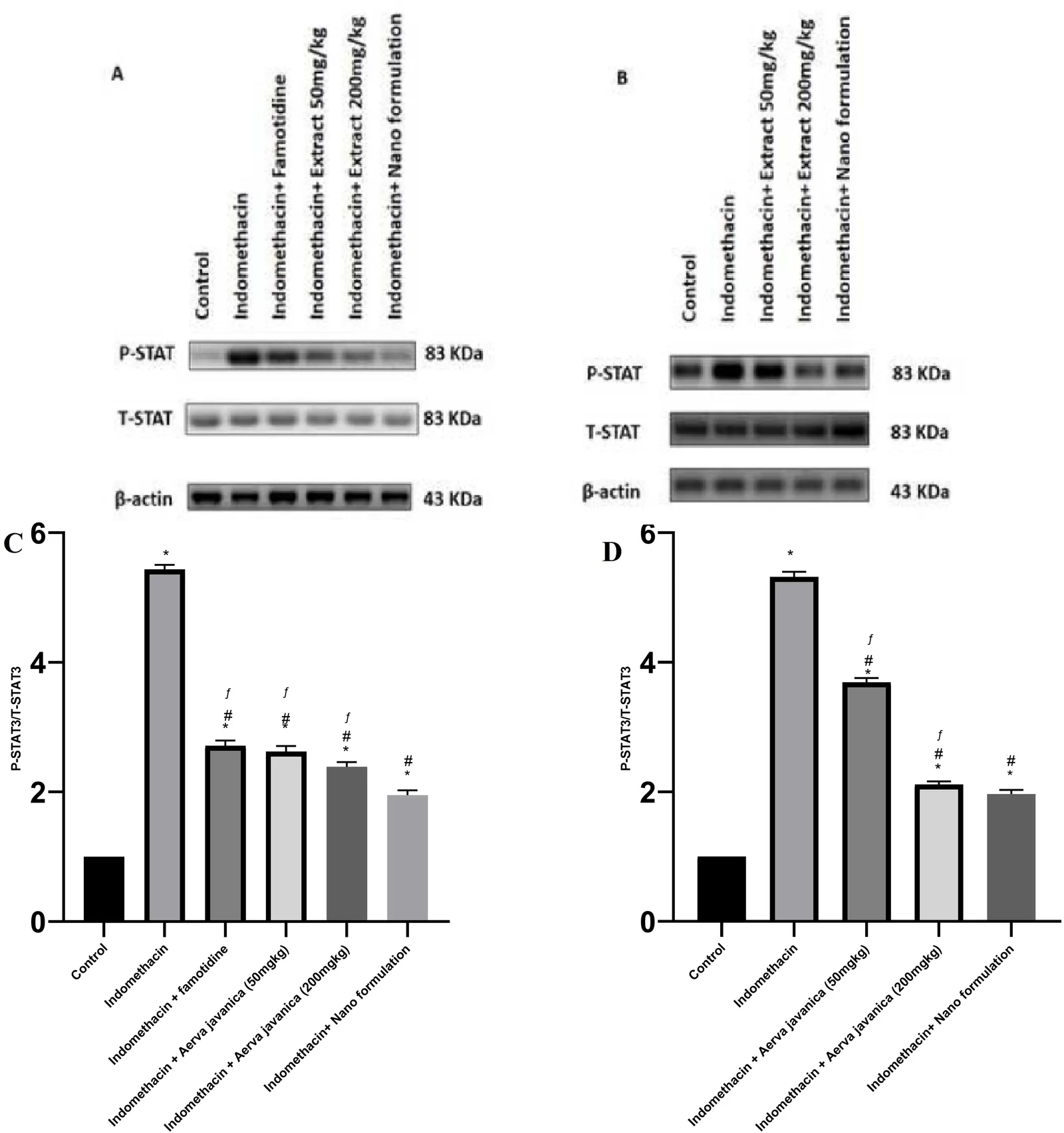
Western blotting was used to identify representative immunological blots of p-STAT-3 and t-STAT-3 in the kidney (B) and stomach (A). C & D: p-STAT-3/t-STAT-3 expression was determined after β-actin normalization. The information was presented as a fold change concerning the control group. With the Image J software, densitometric analysis was carried out. The mean ± SD is shown by the bars. The ANOVA test was utilized to evaluate any significant differences between the research groups, and Tukey’s post hoc test was then performed. * indicates a significant difference (p < 0.05) from the control group. # and ƒ, respectively, denote significant differences between the groups receiving indomethacin alone (p < 0.05) and *A. javanica* nanoparticles (p < 0.05).

### 3.5. Bioinformatic study

#### 3.5.1. Plant-Cpd network.

The identified compounds from *Aerva javanica* are connected in a network called the plant-cpd network (S1 Fig).

#### 3.5.2. Cpd-target network.

The Binding DB and DisGeNET databases were used to identify the targets of the identified compounds; 310 distinct targets were found. According to S2 Fig, the network that was created identified CA12 as having 18 edges, followed by CA7, AKR1B1, CA4, and ADORA1 with 17, 16, 14, and 14 edges, respectively. 337 nodes (compounds and targets) and 798 edges made up this network, which had a network centralization of 0.142 and a typical path length of 3.544.

#### 3.5.3. Target-gastric disorders network.

The common gastrointestinal illnesses (gastritis, gastric ulcer, and associated disorders) were linked to 58 genes. With nine edges, TNF was shown to be the most important gene associated with stomach diseases, followed by the VEGFA gene with seven edges (Fig 13). With 68 nodes and 119 edges, the resulting network had a typical path length of 2.784 and a network centralization of 0.340.

**Fig 13:**
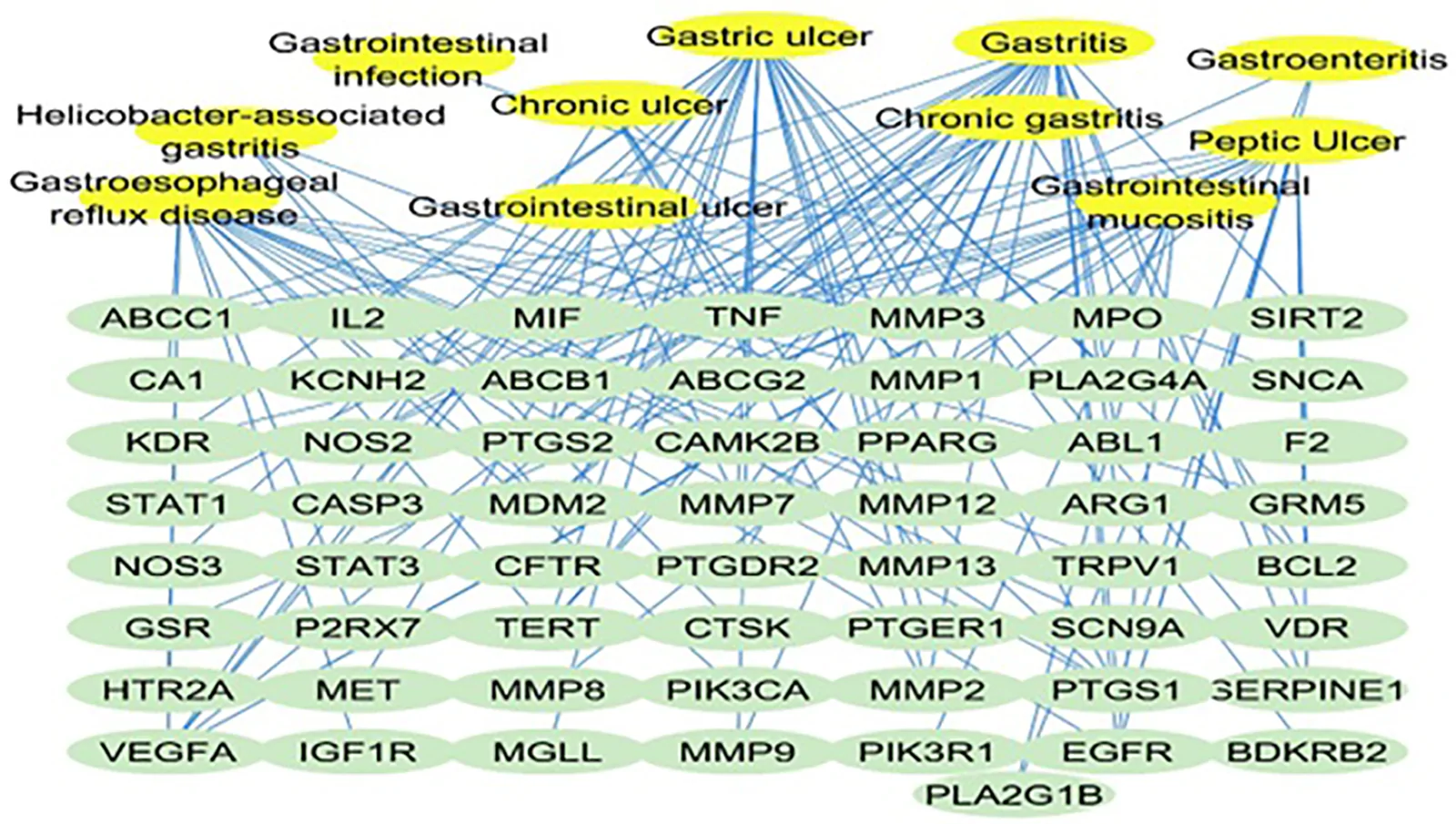
Target-gastric disorders network: A network shows the relationships between common gastric disorders and the targets that have been discovered. Targets are represented by green oval shapes, and stomach issues are represented by yellow oval forms.

#### 3.5.4. Target-nephrotic disorders.

Regarding the network of target nephrotic illnesses, out of the examined data set connected to nephrotic disorders, 73 genes were identified. With 7 and 6 edges, respectively, VEGFA and TNF were the top targets associated with various nephrotic disorders. Network centralization was 0.398, path length 2.798, and node and edge counts of 79 and 148, respectively, comprised the constructed network (Fig 14).

**Fig 14:**
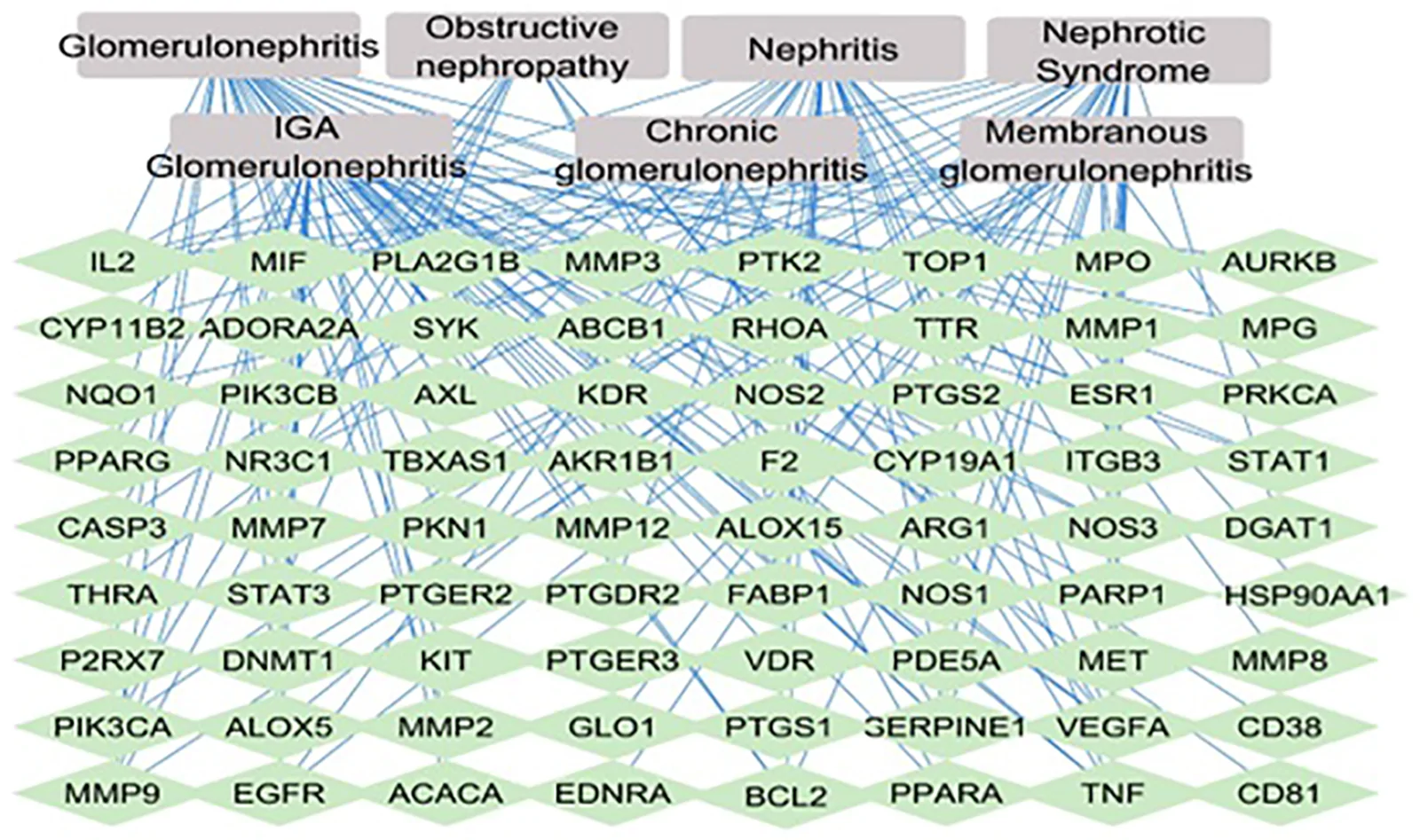
Target-Nephrotic Diseases Network: A network that shows the relationships between nephrotic diseases and targets. Grey rectangles stand in for nephrotic diseases, whereas green ovals indicate objectives.

#### 3.5.5. Cpd-Intersected targets.

Intersected targets are 34 genes (Fig 15A); these genes were identified by 24 metabolites out of the 27 identified metabolites. Compound 21, followed by compound 8, has a high representation of these intersected genes with 9 and 8 edges, respectively. The highly represented genes among the intersected set identified by a large no. of compounds were PTGS2, TNF, PTGS1, IL2, and EGFR with 9,8,8,7,7 edges (Fig 15B), respectively. These target genes are considered the targets with dual nephroprotective and gastroprotective potentials.

**Fig 15:**
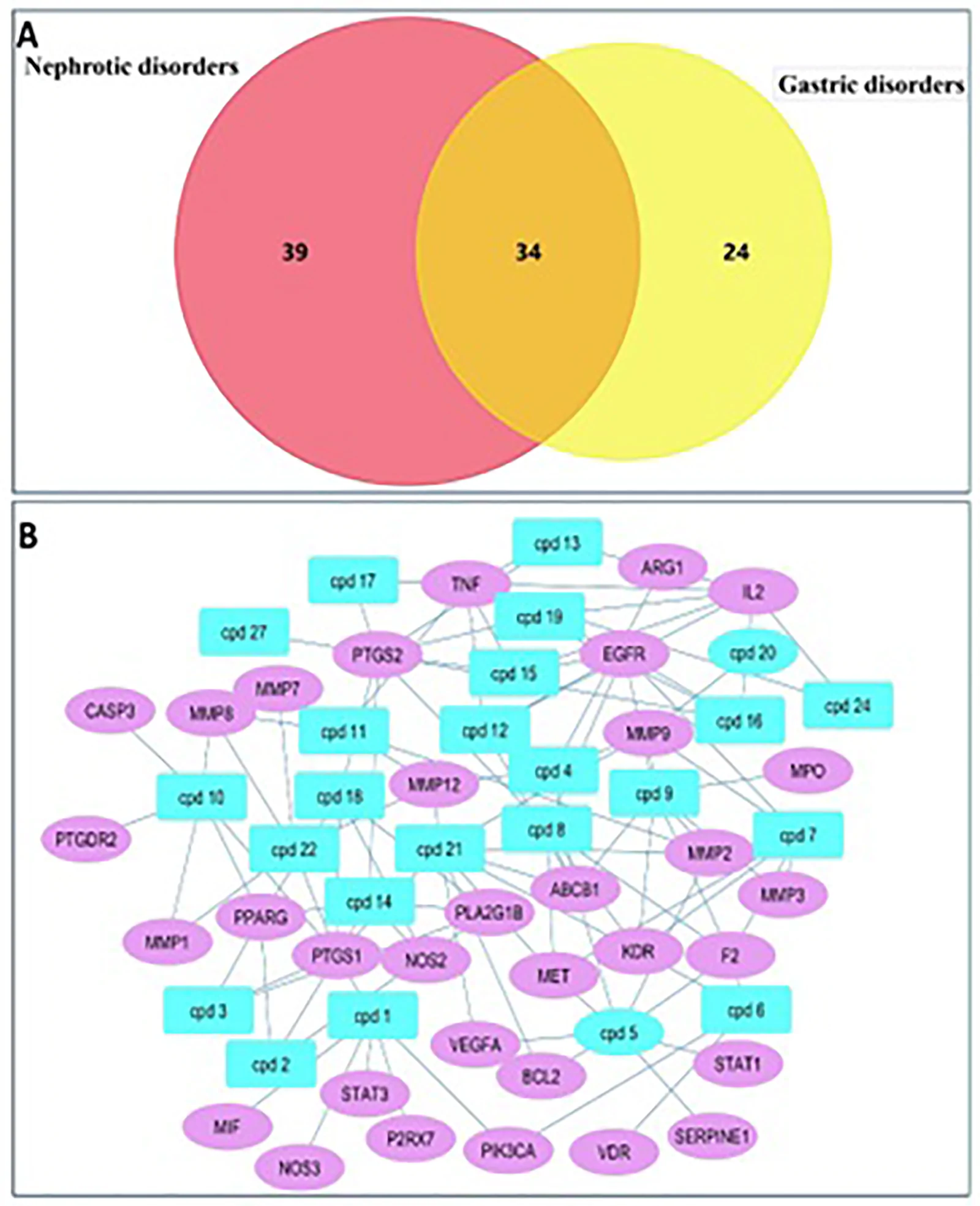
Intersected genes: A) a Venn diagram for the intersected genes of nephrotic disorders and gastric disorders, B) Cpd-intersected genes network; the network illustrates the intersected genes and their connections to the identified compounds.

#### 3.5.6. PPI network.

Using the String database, the PPI network was run to identify the intersecting genes. The node degree analysis of the PPI findings showed that TNF was the top gene. With a local clustering value of 0.768 and an average node degree of 15, the PPI network consists of 33 nodes and 248 edges (Fig 16). The genes with the greatest node degrees, TNF and MMP9, were determined to be 29 and 26, respectively.

**Fig 16:**
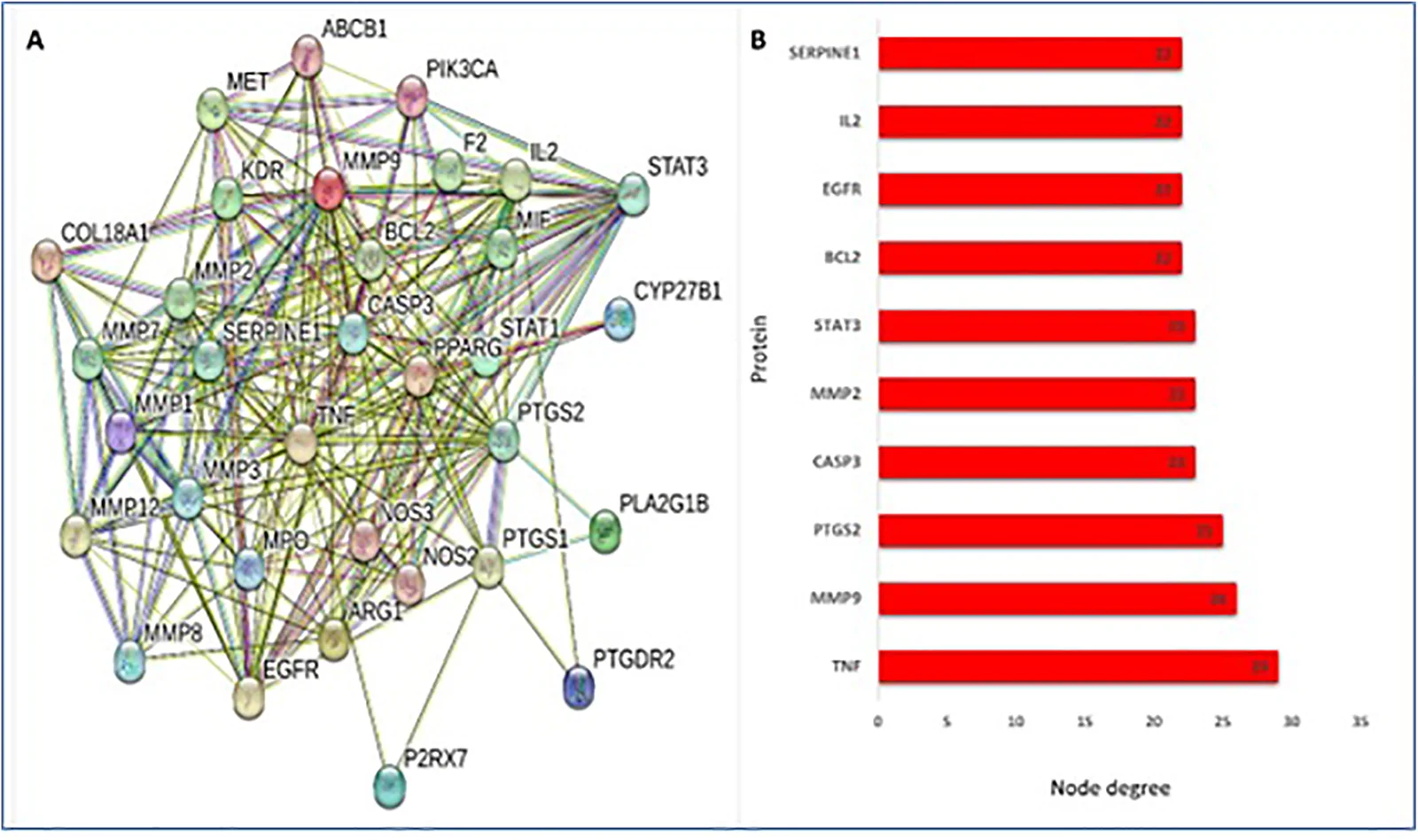
A); Protein-Protein interaction network; B) bar chart for top proteins according to node degree.

#### 3.5.7. Gene enrichment analysis and Gene Ontology (GO).

For the genes that intersected, an enrichment analysis and Gene Ontology study were conducted. The gene ontology was investigated concerning cellular constituents, molecular functions, and biological processes. The KEGG database was used to determine the biological pathways’ enrichment analysis. The databases that were selected were WebGeStalt, SRPlot bioinformatics databases, and the ShinyGO 0.8 database. Collagen metabolic process (GO:0032963), reactive oxygen species response (GO:0000302), oxidative stress response (GO:0006979), and cellular stress response (GO:0062197) were the top biological processes according to the GO (Fig 17).

**Fig 17:**
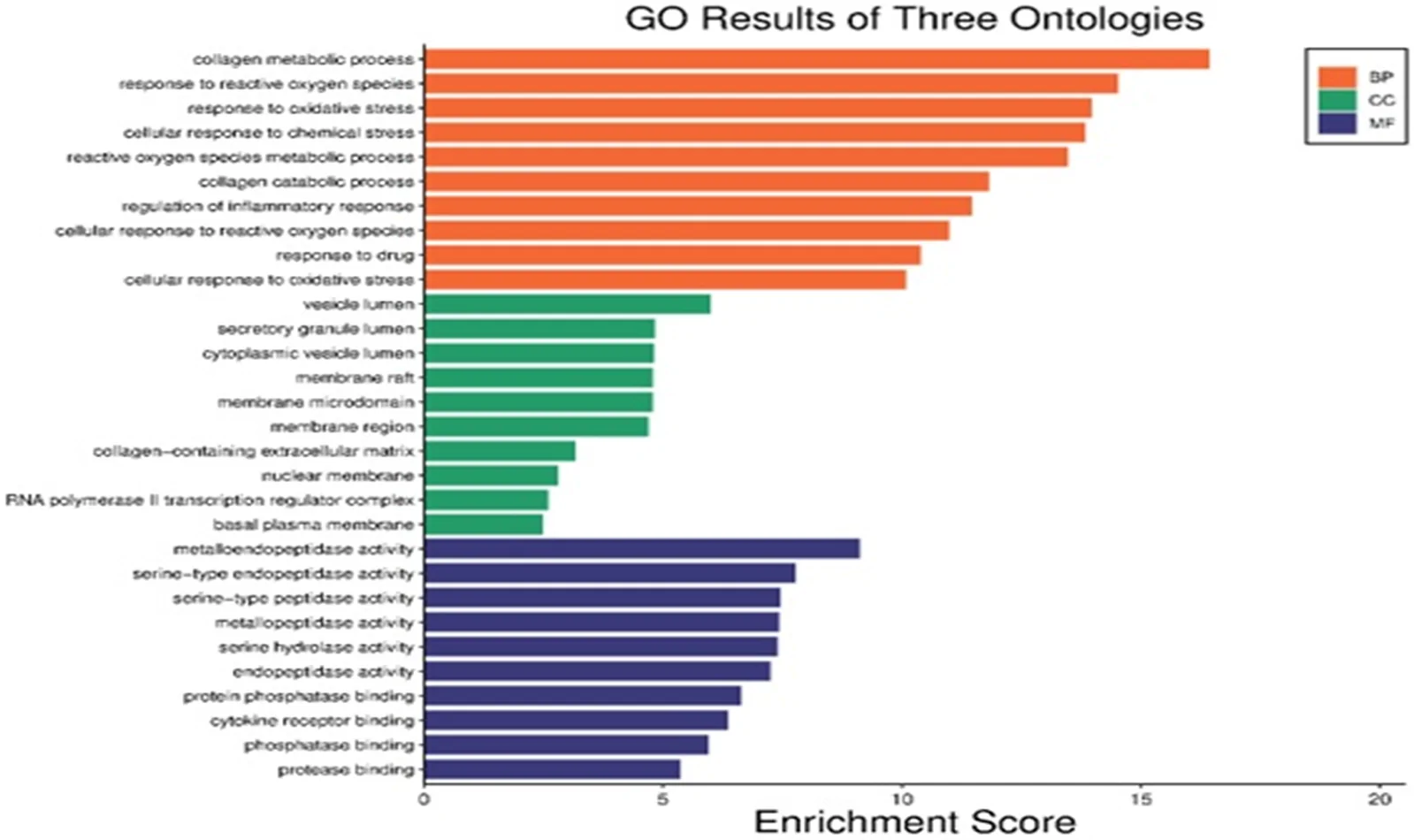
Gene Ontology of the intersecting genes in terms of biological process, cellular component, and molecular function found in Aerva javanica compounds.

Vesicle lumen (GO:0031983), secretory granule lumen (GO:0034774), cytoplasmic vesicle lumen (GO:0060205), and membrane raft (GO:0045121) were the top cellular components according to the GO (Fig 17). Metalloendopeptidase activity (GO:0004222), serine-type endopeptidase activity (GO:0004252), serine-type peptidase activity (GO:0008236), and metallopeptidase activity (GO:0008237) were the GOs in terms of molecular function (Fig 17). Using the enrichment score as a guide, the GO items for each phrase were placed in decreasing order. 117 routes were found using the three datasets. The AGE-RAGE signaling route in diabetes complications (hsa04933) and the HIF-1 signaling pathway (hsa04066) were the two most prominent signaling pathways based on the enrichment score (Fig 18, 19).

**Fig 18:**
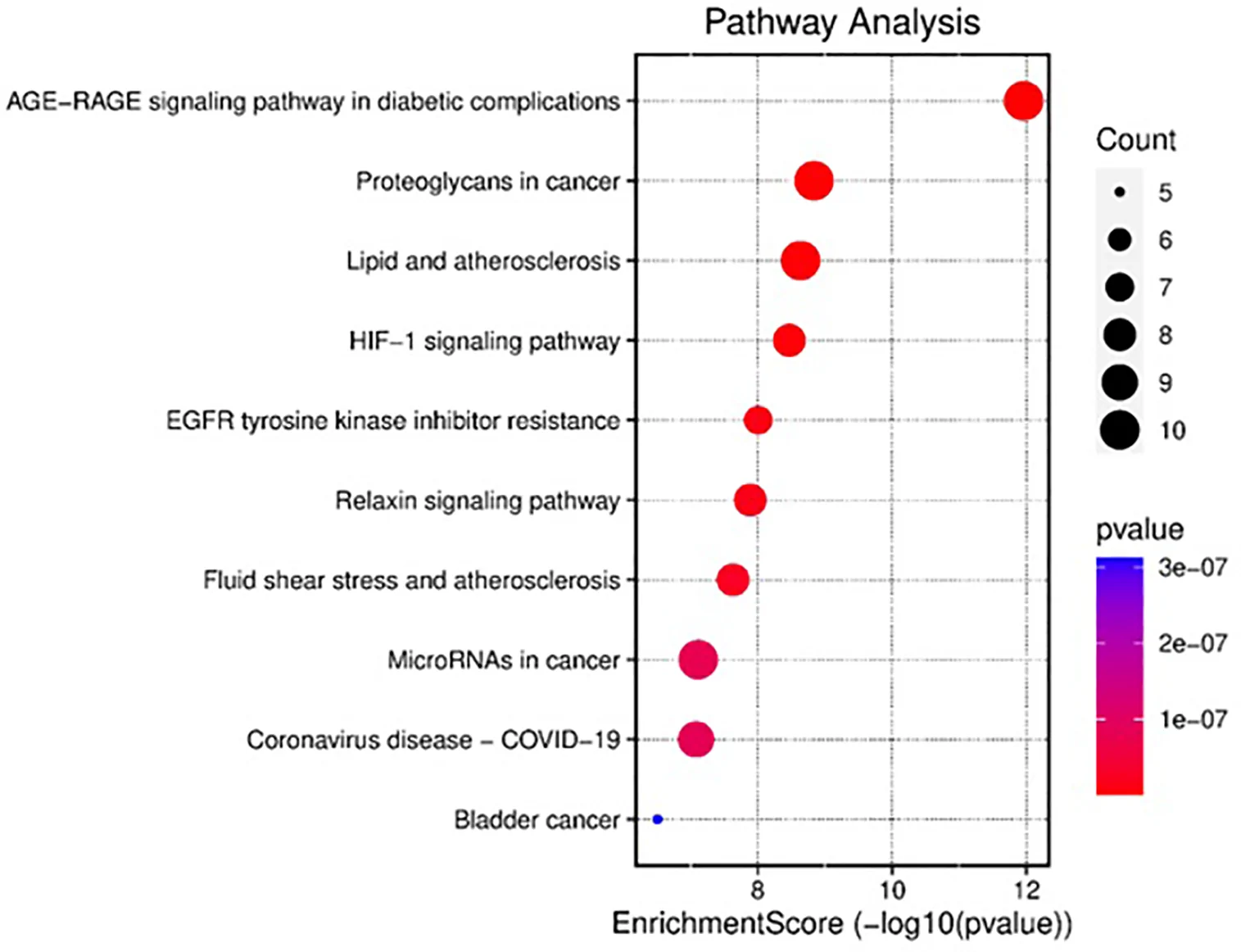
The top 10 biological KEGG pathways based on the score of enrichment.

**Fig 19:**
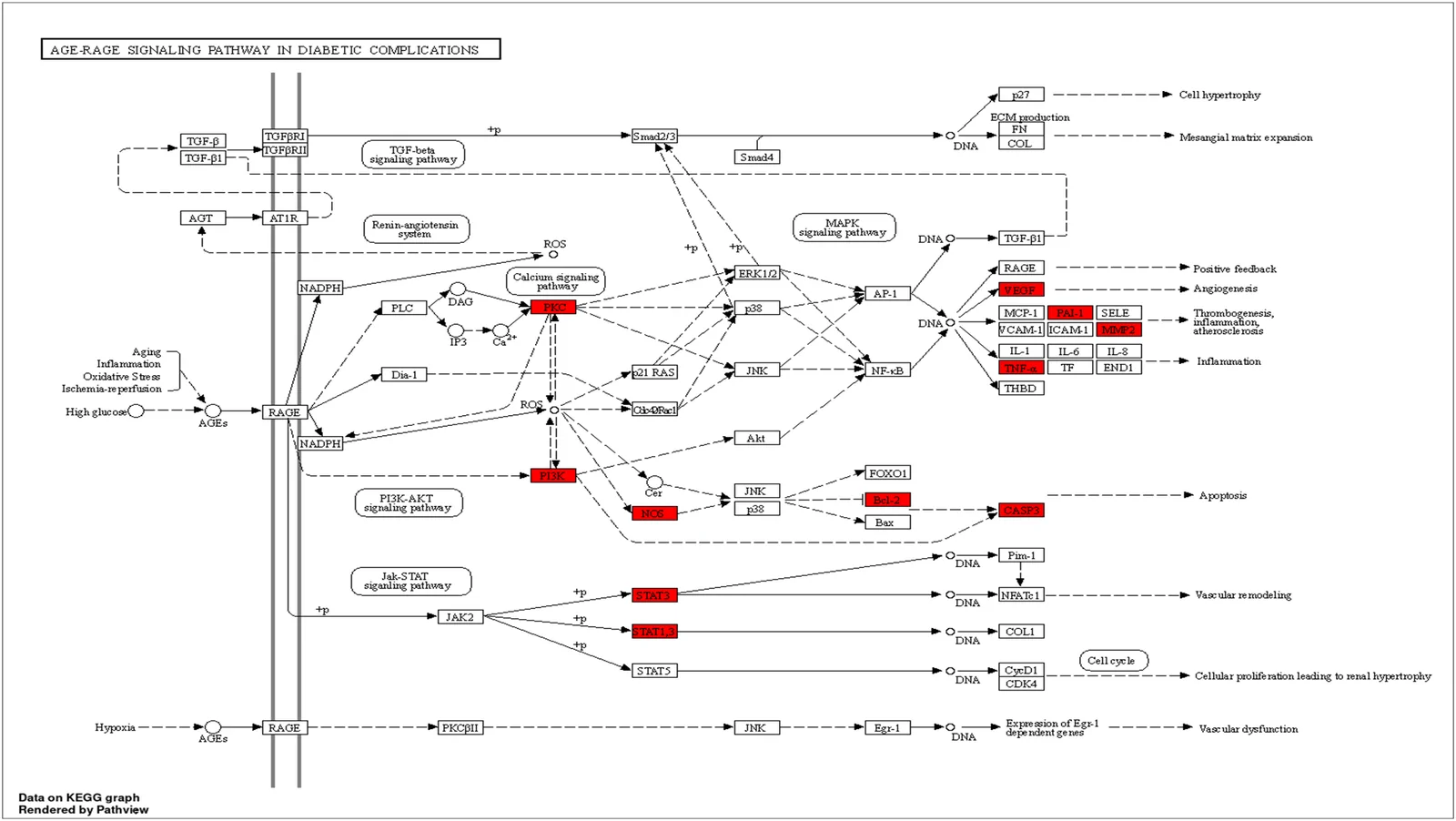
The top biological signaling system with the highest enrichment score in diabetes problems is the AGE-RAGE pathway, in which the intersecting genes are identified as both nephroprotective and gastroprotective.

## 4. Discussion

### 4.1. LC-HR-ESI-MS metabolomic profiling of *Aerva javanica*

As shown in (S1 Table) and (Fig 20), the primary discovered chemical classes in *Aerva javanica* were flavonoids, fatty acids, phenylpropenes, glycosides, and saponins. Based on the metabolomic data, the *Aerva javanica* extract included twenty flavonoid components, one saponin, three fatty acids, one phenyl propene, and one glycoside of hydroxycinnamic acid. Following the chemical formulae C_12_H_16_O_3_ and C_15_H_12_O_5_, the mass ion peaks at m/z 209.11525 [M + H] + (RT, 3.2298 min) and m/z 273.07624 [M + H] + (RT, 3.60795 min) were recognized as elemicin and naringenin, which were previously isolated from Nutmeg and citrus fruits, respectively [51,52]. The molecular formulas C_18_H_30_O_2_ and C_15_H_10_O_6_, which were anticipated, were identified as octadecatrienoic acid based on molecular ion peaks at m/z 279.2324 [M – H] – (RT, 9.038856 min) and m/z 285.03932 [M – H] – (RT, 3.3679 min) [53] and luteolin, which was previously isolated from celery leaves [54]. Furthermore, it was discovered and reported that Epicatechin, which has the chemical formula C_15_H_14_O_6_ with a molecular ion peak at m/z 291.08669 [M + H] + (RT, 3.01856 min), was extracted from *Wisteria floribunda* [55]. Dereplicated Dihydro-5-(tetradecyl 8-oxo) −2-(3H)-furanone, quercetin, and kaempferol 3,4’-O-di-methyl ether from the mass ion peaks at m/z 295.22676 [M – H] – (RT, 8.97907 min) C_18_H_32_O_3_, m/z 303.05022 [M + H] + (RT, 3.071406 min) C15H10O7, m/z 313.07072 [M – H] The compounds C_17_H_14_O_6_ (RT, 6.95188 min) were extracted from *Glycyrrhiza glabra*, *Morus alba*, and *Zingiber zerumbet*, respectively [56]. *Aerva javanica* was found to contain 9,10,11-Trihydroxy-12,15-octadecadienoic acid, in addition to Rhamnetin, which was previously identified from *Ammi visnaga* having a molecular ion peak at m/z 317.06629 [M + H] + (RT, 3.4969967 min) C_16_H_12_O_7_ [57] and *m/z* 327.21671 [M – H] ^–^ (RT, 5.2884233 min) C_18_H_32_O_5_ [58]. Furthermore, the dereplicated data revealed the presence of fourteen additional flavonoid metabolites that had previously been documented in the following species: *Zea mays*, *Cuscula australis*, *Flos chrysanthemi*, *Aesculus hippocastanum*, Grape seeds, Citrus fruits, Wheat grains, and *Quercus ilex*. With molecular formulae C_21_H_20_O_10_, C_21_H_20_O_11_, C_21_H_18_O_12_, C_22_H_22_O_11_, C_21_H_20_O_12_, C_22_H_20_O_12_, C_27_H_28_O_13_, C_30_H_26_O_12_, C_27_H_32_O_14_, C_28_H_30_O_14_, C_27_H_30_O_15_, C_27_H_30_O_16_, C_28_H_32_O_16_, and C39H32O15, these flavonoid metabolites are specifically referred to as Apigenin-7-O-glucoside [59], kaempferol-3-O-β-D-glucoside [60], luteolin-7-O-glucuronide [61], Diosmetin-7-O-glucoside [62], isoquercetin [63], Diosmetin-7-O-glucuronide [64], Apimaysin [65], procyanidin [66], Naringenin-7-O-rutinoside [67], 3’-methoxymaysin [65], isoorientin-2”-O-rhamnoside [68], luteolin-6,8-C-diglucoside [69], Diosmetin-3,8-di-C-glucoside [69], Astragalin; 2,”4”-Bis-O-(4-hydroxycinnamoyl) [70]. They were dereplicated from the molecular ion peaks at *m/z* 433.1135 [M + H] ^+^ (RT, 3.2524 min), *m/z* 447.09216 [M – H] ^–^ (RT, 3.3317 min), *m/z* 461.07197 [M – H] ^–^ (RT, 3.25984 min), *m/z* 461.10769 [M – H] ^–^ (RT, 3.6708 min), *m/z* 463.08702 [M – H] ^–^ (RT, 3.137 min), *m/z* 475.08726 [M – H] ^–^ (RT, 3.7721 min), *m/z* 559.14279 [M – H] ^–^ (RT, 2.7561 min), *m/z* 577.13467 [M – H] ^–^ (RT, 5.47561 min), *m/z* 579.17198 [M – H] ^–^ (RT, 3.5476 min), *m/z* 589.15348 [M – H] ^–^ (RT, 3.00939 min), *m/z* 593.15077 [M – H] ^–^ (RT, 3.1452 min), *m/z* 609.14595 [M – H] ^–^ (RT, 3.00824 min), *m/z* 623.16155 [M – H] ^–^ (RT, 3.205233 min), and *m/z* 739.1673 [M – H] ^–^ (RT, 6.51615 min) respectively. Additionally, oleanolic acid, a different fatty acid that was previously isolated from Olea europea, was reproduced from the Aerva javanica extract. The mass ion peaks for the proposed molecular formulae C_30_H_48_O_3_ are located at m/z 455.35196 [M – H] – (RT, 11.228853 min) [71]. Lastly, sinapoyl-D-glucoside was dereplicated at *m/z* 385.11269 [M – H] ^–^ (RT, 2.56742 min) for the suggested molecular formulas C_17_H_22_O_10_ and reported to be isolated from *Brassica oleracea* [72].

**Fig 20:**
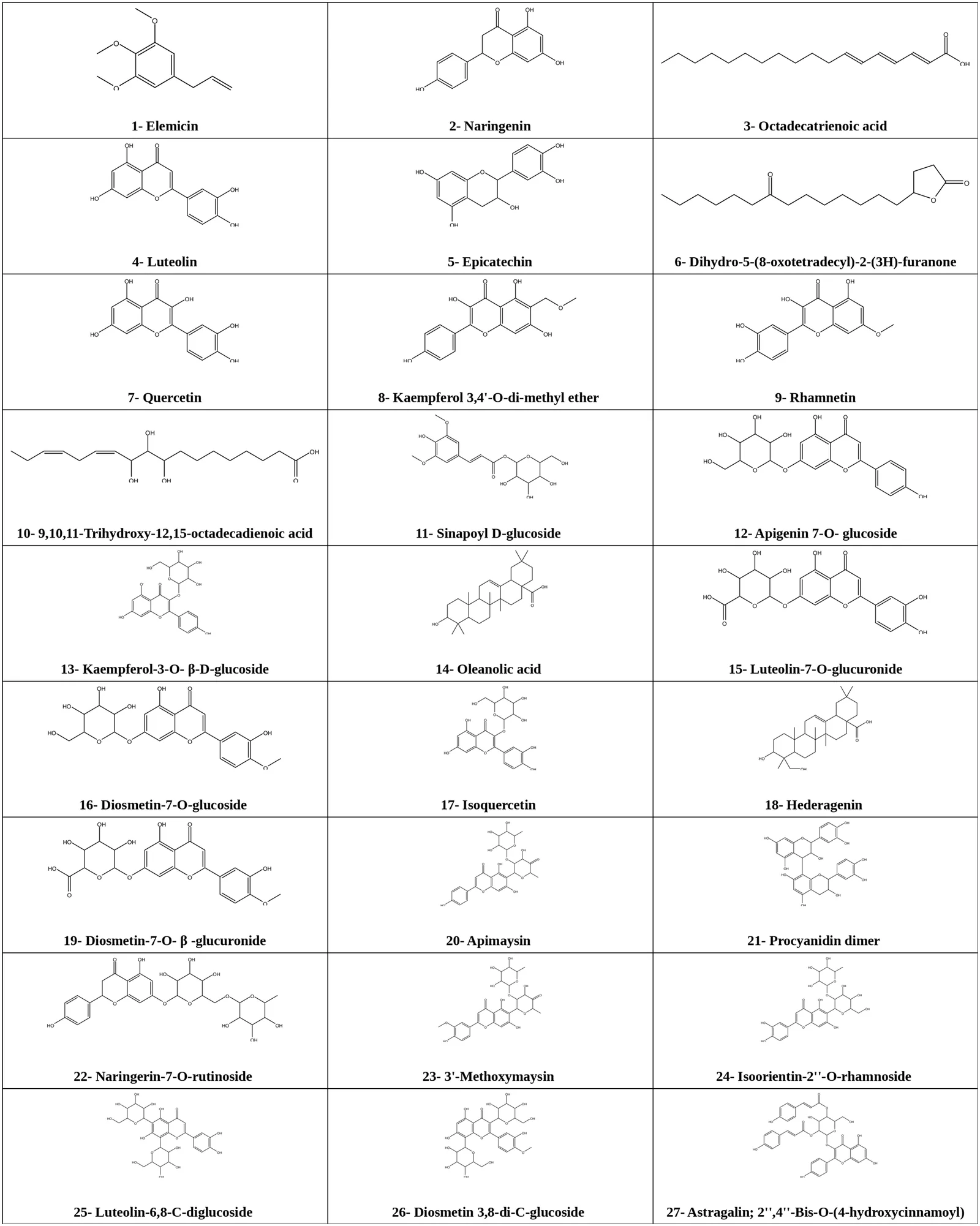
Components from the methanolic extract of *Aerva javanica* have been duplicated.

### 4.2. Characterization of the synthesized *Aerva javanica* SNPs

SNPs were synthesized using the methanolic extract of *Aerva javanica*. The initial method to verify SNP synthesis in the reaction mixture was to look for a color shift. SNPs were created after 10 minutes of incubation at 60 °C, as shown by the appearance of a reddish-brown color [73]. Similar conclusions were reported by Jaidev. Because of the surface plasmon excitation and vibrations [74], SNPs have a reddish-brown color. The quantity of silver ions in the silver nitrate solution drops when *Aerva javanica* methanolic extract is introduced [75], resulting in the formation of SNPs and a color change. A theory implies that the presence of secondary metabolites in plants generates a redox reaction that might be leveraged to aid in the manufacture of nanoparticles, even if the exact mechanism underpinning the formation of SNPs is yet unclear. These metabolites are responsible for the production and stability of the biosynthesized nanoparticles [76]. Ag + ion levels were shown to be declining in the UV-visible spectrum, and this finding was corroborated by an absorbance peak at 415 nm. The size and shape of the SNPs may indicate the absorbance peak. There is a linear correlation between the peak intensity and the nanoparticle size. The present study’s findings confirm that spherical nanoparticles were produced, with a mean size assessed by TEM to be 20.59 nm. FTIR analysis identified the active components in the plant extracts, which are known to interact with Ag+ through functional groups and aid the reduction to Agº nanoparticles [77]. Unfortunately, additional characterization of nanoparticles (e.g., Zeta potential) were not available.

### 4.3. Nephroprotective and gastroprotective effect of *Aerva javanica* methanolic extract

The design, production, and manipulation of materials at the nanoscale level to get unique features that may be appropriately used for the necessary applications is known as nanotechnology [78]. Nanoparticles (NPs) are small particles with at least one dimension that range in size from 1 to 100 nm. Because of their physicochemical characteristics, which include catalytic activity, electrical and thermal conductivity, light absorption, melting point, wettability, quantum confinement, and dispersion without agglomeration and scattering, NPs have become more prominent in technological advancements [79]. The physicochemical characteristics, tiny size, and high monodispersity of metallic nanoparticles (MNPs) have drawn a lot of attention [80]. Due to their special qualities, MNPs have been discovered to have potential uses in targeted drug delivery, cancer treatment, biosensing, optical devices, electronic, magnetic, catalysis, water treatment, and antimicrobial applications [81]. MNPs, especially those made of silver has garnered a lot of interest and have become a novel class of NPs [81]. Silver nanoparticles, in particular, have garnered a lot of interest and have become a new class of NPs. In particular, silver nanoparticles (AgNPs) have drawn particular attention because of their applications in fields including composite fibres, biosensor materials, waste water treatment, pollution remediation, food packaging materials, food storage containers, cosmetics, and water purifiers [82].

The kidney and stomach are vital organs that are susceptible to the deleterious impacts of NSAIDs [83]. Indomethacin exerts a non-specific inhibitory effect on the synthesis of prostaglandins by targeting the COX enzyme. Nevertheless, the previous assertion that the adverse effects caused by indomethacin are exclusively attributed to the inhibition of COX has been discredited [84]. Multiple investigations have revealed that indomethacin elicits additional effects within the tissue, including the initiation of oxidative stress and impairment of mitochondrial function [7,8]. Hence, it has been hypothesized that these aforementioned effects, alongside the inhibition of COX, play a role in the manifestation of organ dysfunction caused by indomethacin [85]. This study, for the first time, aimed to assess the potential protective properties bestowed by *A. javanica* in mitigating indomethacin-induced acute renal and gastric toxicity in rats. Furthermore, an investigation was conducted to explore the potential mechanisms underlying the renal and gastroprotective effects by assessing oxidative stress-induced inflammation as well as molecular signaling pathways.

Indomethacin exerts its effect by inhibiting the activity of COX, resulting in a reduction in the level of prostaglandins. Moreover, it exerts a suppressive effect on mucus and HCO_3_ − secretion as well as blood flow within the mucosal layer. These effects also make substantial contributions to alterations in microvascular structures [86]. The gastric ulcer index was employed as a measure of gastric injury. In this particular scenario, it was observed that the rats subjected solely to indomethacin treatment exhibited a notable increase in ulcer index. The administration of two doses of *A. javanica* or nano formulation treatment exhibited a notable improvement in gastric mucosal damage. This was observed through a significant decrease in the ulcer index. Our observations align with the results of prior investigations, which have likewise exhibited the occurrence of gastric mucosal damage following indomethacin treatment [87,88].

To enhance our understanding of renal injury caused by indomethacin in our rat model, we conducted an assessment of markers associated with tubular and glomerular damage. KIM-1 is a transmembrane protein that is found to be expressed at relatively low levels in the normal kidney by the proximal tubular epithelial cells [89]. It is robustly induced in response to acute injury of the renal tubules. Henceforth, it is regarded as a discerning indicator of tubular impairment [90]. Elevated concentrations of serum creatinine and BUN serve as indicators of glomerular injury [91]. The experimental findings demonstrate the induction of KIM-1 protein expression after indomethacin treatment. Furthermore, we have observed heightened levels of serum creatinine and BUN. This finding provides further support to the existing evidence indicating that indomethacin induces acute renal damage, which is closely associated with gradual histopathological changes [2]. The administration of either low or high doses of *A. javanica* or the nano-formulation treatment resulted in a significant enhancement of renal functions. Our data revealed a notable suppression in serum KIM-1, serum creatinine, and BUN levels, with the most favorable outcomes observed in the rats treated with the nano-formulation. It is important to report that administration of nanoformulation did not cause elevation in renal functions (BUN, creatinine, KIM-1). The absence of renal injury markers and histopathology in the nanoparticle-only group suggests acceptable biocompatibility under the tested conditions.

During the process of ATP synthesis via mitochondrial respiration, ROS are generated [92]. The dysregulation of free radical generation and scavenging capability gives rise to oxidative stress, a pivotal factor in the development of gastric ulcers. These ROS induce harm to the gastric tissue through the impairment of membranes and cellular macromolecules, including lipids, proteins, carbohydrates, and nucleic acids [93]. Prior research has indicated that ROS-mediated oxidative stress plays a significant role in the onset and perpetuation of indomethacin-induced AKI [7, 85, 90, 94]. NSAIDs, such as indomethacin, have been observed to elicit an elevation in oxidative stress levels through the augmentation of lipid peroxidation processes [8]. The infiltration of neutrophils in response to the administration of NSAIDs has been observed in the stomach and kidney. Activated neutrophils, through their metabolic processes, produce ROS [3,95]. To evaluate the impact of the extract on the redox status, we measured MDA levels and SOD levels in gastric and renal tissues. MDA levels, a byproduct of lipid peroxidation that serves as a prevalent marker for assessing the presence of oxidative stress in cellular and tissue impairment [96]. In this investigation, the levels of tissue MDA exhibited a significant increase in rats with renal and gastric damage, as compared to the control group. Contrarily, administration of extract at doses of 50 mg/kg and 200 mg/kg resulted in a notable reduction of elevated MDA levels in both ulcerated gastric tissue and the kidney. The ability of the extract to scavenge ROS leads to a reduction in MDA levels, thereby aiding in the mitigation of oxidative damage in the gastrointestinal tract and the prevention of acute kidney injury induced by ROS. Furthermore, the application of nano formulation treatment yielded a significantly greater decrease in the concentrations of MDA within both the gastric and renal tissues.

The body employs endogenous antioxidants to counteract the detrimental impacts of continuously generated ROS on the body [97,98]. SOD is a naturally occurring antioxidant that plays a crucial role in maintaining the integrity of mucosal tissues and safeguarding the gastric mucosa against harm caused by free radicals [99]. Additionally, SOD plays a crucial role in safeguarding the kidney from heightened levels of ROS that trigger AKI. According to prior investigations, the administration of indomethacin has been observed to decrease the levels of SOD in both stomach and kidney tissues [100]. In our investigation, we observed a depletion of SOD in the gastric and renal tissues of rats afflicted with gastric ulcers and AKI. The application of both doses of *A. gavanica* resulted in a notable elevation in SOD levels. The observed elevation in SOD activity indicates that the extract exhibits a protective effect against the onset of AKI and gastric ulcer. This protection is likely achieved through the reduction of ROS-induced oxidative stress and the enhancement of the body’s antioxidant defense mechanisms. Furthermore, the administration of the nano formulation exhibited a significantly greater augmentation in the SOD concentration. The scavenging activity of ROS exhibited by the extract can be attributed to the presence of flavonoids and phenolic compounds within its composition.

NF-κB is a key regulator in immune and inflammatory pathways, including gastric ulcers [101] and AKI [102,103]. It is reported to be synthesized rapidly in response to ROS-associated cellular damage, with its activation involving degradation of the IκB-α protein. Upon activation, the NF-κB complex is thought to translocate from the cytoplasm to the nucleus, where it may induce TNF-α and IL-1β [104,105]. In the present study, gastric and renal injury was accompanied by increased levels of phosphorylated NF-κB p65 protein relative to the control group, consistent with activation of the IκB/NF-κB pathway. However, extract administration was associated with a downregulation of the IKκB/NF-κB pathway, accompanied by a reduction in phosphorylation and degradation of IκB-α. These findings suggest, that the extract modulates NF-κB activation. Previous research has indicated that antioxidants such as polyphenolics may hinder NF-κB activation by inhibiting IκB-α phosphorylation [106] raising the possibility that the extract’s ROS-scavenging capacity contributes to its inhibitory association with NF-κB activation.

TNF-α is a key biomarker as it has a central pro-inflammatory cytokine driving the pathogenesis of both indomethacin-induced gastric ulcers and AKI. In gastric injury, indomethacin inhibits COX-1/prostaglandin E2, disrupting mucosal barriers and triggering neutrophil infiltration and apoptosis. TNF-α amplifies this via activating the NF-κB pathway. This route is essential for controlling the transcription of different adhesion molecules. As a result, the increase in these molecules helps the infiltration of inflammatory cells, which has been seen in rats given indomethacin. Moreover, in indomethacin-induced AKI, TNF-α mediates tubular damage by promoting oxidative stress, endothelial dysfunction, and leukocyte recruitment in the renal interstitium, contributing to cast formation, apoptosis, and fibrosis—effects observed at 12 hours post-challenge, aligning with the study’s renal subgroup timing. IL-1β, a cytokine known for its pro-inflammatory properties, plays a vital role in controlling several genes linked to the inflammatory response and the resulting tissue damage [107,108]. The modulation or inhibition of the release of inflammatory cytokines shows potential as a therapeutic strategy for mitigating gastric ulcers and kidney injury. The extract demonstrated the capacity to hinder the infiltration of inflammatory cells in the stomach and kidney tissues at both dosage levels. Furthermore, it resulted in notable decreases in the concentrations of TNF-α and IL-1β in the mucosal epithelium and renal tissues. These data indicate that the extract has an anti-inflammatory effect in this particular situation.

Prior research has indicated that TNF-α can induce the overexpression of iNOS, leading to increased production of nitric oxide (NO) in a model of indomethacin-induced jejunoileitis [109]. The presence of NO contributes to the development of stomach ulcers by initiating the formation of peroxynitrite radicals (ONOO-), which cause cell toxicity, protein tyrosine nitration, synthesis of hydroxyl radicals, and subsequent tissue damage [31]. The production of NO by the three isoforms of NOS in the kidney is crucial for maintaining renal function. All three isoforms of NOS are present in the kidney, with the endothelial isoform located in the renal vasculature and the neuronal (constitutive) isoform found in the macula densa. The release of iNOS is triggered by cytokines, lipopolysaccharide, and ischemia/reperfusion (I/R) [110,111]. Elevated levels of NO produced by iNOS have been linked to kidney dysfunction and damage observed in renal I/R, endotoxic shock, and hemorrhagic shock situations [112–114]. Various studies have shown that inhibiting iNOS expression, reducing iNOS activity, eliminating iNOS, or removing NO can enhance NO-induced kidney injury [112,115]. The present study showed that indomethacin led to an increase in the expression of iNOS inside the stomach & renal tissues associated with an increase in TNF-α. The extract had an inhibitory effect on the expression of iNOS leading to diminished NO release, which is recognized to exacerbate stomach mucosal and renal damage. Additionally, the application of a nano formulation prior to treatment resulted in a further reduction in the levels of iNOS in the stomach & kidney.

The JAK/STAT3 and NF-κB signaling pathways have been reported in the literature to be important contributors to renal inflammation and gastric ulceration. Based on prior studies, cytokine stimulation is thought to activate JAK, which may subsequently be associated with phosphorylation of STAT3. Phosphorylated STAT3 has been reported to translocate to the nucleus, where it may facilitate transcription of genes associated with inflammation [116]. Moreover, the JAK/STAT3 complex has been reported to be associated with NF-κB activation through phosphorylation of its p65 subunit, a process implicated in the transcription of inflammation-related genes. The interplay between these pathways has been proposed to be associated with exacerbation of the inflammatory response in the kidneys and stomach, and, in turn, with tissue damage [117]. In consistence with these reports, our study observed that the phosphorylation of STAT3 was inhibited in response to indomethacin-induced toxicity. This inhibition was reversed in parallel with extract administration in a dose-dependent way. Althogh the proposed ROS–NF-κB–JAK/STAT signaling axis was not directly investigated experimentally in the present study, the observed results of the increased STAT3 in indomtheacin treated rats along with the previous reports supports that the inhibition of ROS–NF-κB–JAK/STAT signaling may be associated with the potential protective effect of *A. javanica*.

### 4.4. Bioinformatic study

The bioinformatics study supported the evaluation of the nephroprotective and gastroprotective effects of the *Aerva javanica* extract. The study identified compound 21 (Procyanidin dimer) and compound 8 (Kaempferol 4'-O-methyl ether) to have a high association with the dual activity genes. The determined biological process term of gene ontology was the collagen metabolic process. Reaction to reactive oxygen species and reaction to oxidative stress were the top signaling pathways, and they were probably associated with the extract’s antioxidant activity. The AGE-RAGE signaling route in diabetes complications and the HIF-1 signaling pathway were the theoretically identified biological mechanisms that need future confirmatory studies. One of the key mechanisms in diabetic complications that inhibits inflammatory mediators like MMP2 and TNF-α is the AGE-RAGE signaling pathway. Together with other genes found in the same pathway, BCL-2 and CASP-3 reflected the pathway’s inhibition of cellular death. By blocking vascular tones, iNOS and eNOS mediated the suppression of oxidative stress via the HIF-1 pathway. The TNF gene, which is represented as TNF-α in the AGE-RAGE signaling pathway in diabetic complications, has the greatest node degree in the PPI network, which may reflect a potential role in oxidative stress inhibition. All the network pharmacology studies are predictive and offer a guideline for possible target genes and pathways, and need practical confirmatory evaluation in the future.

## 5. Conclusion

Excitingly, our study has revealed the remarkable potential of *A. javanica* extract and its green-synthesized silver nanoparticle formulation in significantly ameliorating gastric ulcers and AKI induced by indomethacin administration. This protective effect was accompanied by attenuation of oxidative stress, improvement of histopathological features, and modulation of inflammatory markers, including NF-κB p65 and STAT-3 expression. The nanoparticle formulation exhibited enhanced protective efficacy compared to the crude extract, supporting its potential as an optimized delivery system for bioactive phytoconstituents. Overall, these results provide experimental evidence supporting the gastroprotective and nephroprotective potential of A. javanica, particularly in nanoparticle form.

## Supporting information

S1 Table Dereplicated compounds from the methanolic extract of *Aerva javanica.* (DOCX)

S1 Fig Plant-cpd network; a network illustrates the identified compounds from the plant (*Aerva javanica*). (JPG)

S2 Fig Cpd-target network; a network illustrates the connections between the identified compounds of *Aerva javanica* and their annotated genes. (JPG)
