# Supplementary material for: Nephroprotective and Gastroprotective potential of Aerva javanica (L.) green synthesized nanoparticles, supported by Molecular Networking
Source: PLoS One. 2026 Nov 5;21(11):e0358843. doi: 10.1371/journal.pone.0358843 (PMC13739114; doi:10.1371/journal.pone.0358843)
Supplement: Supplementary file 1 [file pone.0358843.s001.docx]

**S1 Table. Dereplicated compounds from the methanolic extract of *Aerva javanica*.**

| **No.** | **(+) ESI-MS**  **(M/Z)** | **R.T.**  **(min)** | **Ionization (ESI+/ESI-)** | **M.wt.** | **Compounds Identified** | **Molecular formula** | **Exact mass difference** | **Biological Source** | **References** |
| --- | --- | --- | --- | --- | --- | --- | --- | --- | --- |
| 1 | 209.11525 | 3.2298767 | [M + H] ^+^ | 208.10798 | Elemicin | C12H16O3 | 1.02 | Nut meg,  Sweet basil,  Carrot,  Pepper,  Japanese star anise,  Tarragon,  Sweet bay | [1] |
| 2 | 273.07624 | 3.6079567 | [M + H] ^+^ | 272.06897 | Naringenin | [C15H12O5](https://pubchem.ncbi.nlm.nih.gov/search/#query=C15H12O5) | 0.495 | Citrus fruits (Orange and grape fruit),  Tomato | [2] |
| 3 | 279.2324 | 9.0388567 | [M + H] ^+^ | 278.22513 | Octadecatrienoic acid | [C18H30O2](https://pubchem.ncbi.nlm.nih.gov/search/#collection=compounds&query_type=mf&query=C18H30O2&sort=mw&sort_dir=asc) | 0.13 | Catalpa species,  *Aerva javanica* | [3] |
| 4 | 285.03932 | 3.3679 | [M − H] ^−^ | 286.0466 | Luteolin | [C15H10O6](https://pubchem.ncbi.nlm.nih.gov/search/#query=C15H10O6) | 1.14 | Celery, parsley, broccoli, onion leaves, carrots, peppers, cabbages, apple skins, and chrysanthemum flowers | [4] |
| 5 | 291.08669 | 3.01856 | [M + H] ^+^ | 290.07941 | Epicatechin | [C15H14O6](https://pubchem.ncbi.nlm.nih.gov/search/#query=C15H14O6) | 0.37 | *Wisteria floribunda,*  *Cassia grandis,*  *Acacia catechu,*  *Rhoicissus tridentata,*  *Lysidice rhodostegia,*  *Juniperus,*  *Communis var. depressa,*  *Livistona chinensis,*  *Cassia sieberiana,*  *Thespesia populnea,*  *Chaenomeles speciosa.* | [5] |
| 6 | 295.22676 | 8.9790733 | [M − H] ^−^ | 296.23403 | Dihydro-5-(8-oxotetradecyl)-2-(3H)-furanone | [C18H32O3](https://pubchem.ncbi.nlm.nih.gov/search/#query=C18H32O3) | 1.07 | *Glycyrrhiza uralensis* | [6] |
| 7 | 303.05022 | 3.0714067 | [M + H] ^+^ | 302.04295 | Quercetin | [C15H10O7](https://pubchem.ncbi.nlm.nih.gov/search/#query=C15H10O7) | 0.3 | *Morus alba,*  *Camellia sinensis,*  *Moringa oleifera,*  *Calamus scipionum,*  *Apium graveolens* | [6] |
| 8 | 313.07072 | 6.95188 | [M − H] ^−^ | 314.07799 | [Kaempferol 3,4'-di-O-methyl ether](http://www.molbase.com/en/cas-20869-95-8.html) | C17H14O6 | 1.05 | *Zingiber zerumbet* | [7] |
| 9 | 317.06629 | 3.4969967 | [M + H] ^+^ | 316.05901 | Rhamnetin | [C16H12O7](https://pubchem.ncbi.nlm.nih.gov/search/#query=C16H12O7) | 0.01 | *Ammi visnaga,*  *Lycium ruthenicum, Thymus nummularius, Persicaria hydropiper,*  *Moringa oleifera,*  *Ziziphus mistol,*  *Artemisia campestris,*  *Haplopappus multifolius, Rhaponticoides iconiensis[, and Salvia* | [8] |
| 10 | 327.21671 | 5.2884233 | [M − H] ^−^ | 328.22399 | 9,10,11-Trihydroxy-12,15-octadecadienoic acid | [C18H32O5](https://pubchem.ncbi.nlm.nih.gov/search/#collection=compounds&query_type=mf&query=C18H32O5&sort=mw&sort_dir=asc) | 0.91 | *Capsicum annuum,*  *Cestrum parqui*,  *Aerva javanica* | [9] |
| 11 | 385.11269 | 2.5674267 | [M − H] ^−^ | 386.11997 | Sinapoyl D-glucoside | [C17H22O10](https://pubchem.ncbi.nlm.nih.gov/search/#collection=compounds&query_type=mf&query=C17H22O10&sort=mw&sort_dir=asc) | 1.33 | *Brassica oleracea,*  cauliflower | [10] |
| 12 | 433.1135 | 3.2524933 | [M + H] ^+^ | 432.10623 | Apigenin 7-O-glucoside | C21H20O10 | 0.63 | *Cosmos bipinnatus, Zinnia elegans,*  *Cynara scolymus* | [11] |
| 13 | 447.09216 | 3.3317233 | [M − H] ^−^ | 448.09944 | Kaempferol-3-O- β-D-glucoside | [C21H20O11](https://pubchem.ncbi.nlm.nih.gov/search/#query=C21H20O11) | 0.56 | *Cuscuta australis* | [12] |
| 14 | 455.35196 | 11.228853 | [M − H] ^−^ | 456.35924 | Oleanolic acid | [C30H48O3](https://pubchem.ncbi.nlm.nih.gov/search/#query=C30H48O3) | 1.1 | *Olea europaea* | [13] |
| 15 | 461.07197 | 3.25984 | [M − H] ^−^ | 462.07925 | Luteolin-7-O-glucuronide | [C21H18O12](https://pubchem.ncbi.nlm.nih.gov/search/#query=C21H18O12) | 0.58 | *Codariocalyx motorius, Ixeris dentata,*  *Perilla frutescens,*  *Remirea maritima* | [14] |
| 16 | 461.10769 | 3.6708133 | [M − H] ^−^ | 462.11496 | Diosmetin-7-O-glucoside | [C22H22O11](https://pubchem.ncbi.nlm.nih.gov/search/#query=C22H22O11) | 1.255 | *Flos Chrysanthemi,*  *Galium verum* | [15] |
| 17 | 463.08702 | 3.13716 | [M − H] ^−^ | 464.0943 | Isoquercetin | [C21H20O12](https://pubchem.ncbi.nlm.nih.gov/search/#query=C21H20O12) | 1.18 | *Aesculus* hippocastanum *(*Horse chestnut*),* Ruscusaculeatus *(Butcher’s Broom), Ginkgo biloba* (Ginkgo)*, Hypericum perforatum* (St. John’s Wort)*, Calendula officinalis* (Pot marigold)*, Arctostaphylos uva ursi* (Uva ursi), Equisetum arvense (Horsetail)*, Glycyrrhiza glabra* (Licorice)*,* *Foeniculum vulgare* (Fennel)*, Aspalathus linearis* (Rooibos)*, Humulus lupulus* (Hops), *Tussilago farfara* (Coltsfoot), *Drosera rotundifolia* (Sundew), *Vaccinium myrtillus* (Bilberry), *Bupleurum chinense* (Bupleurum), *Fouquieria splendens* (Ocotillo), and *Morus spp*. | [16] |
| 18 | 471.34691 | 9.1365467 | [M − H] ^−^ | 472.35419 | Hederagenin | [C30H48O4](https://pubchem.ncbi.nlm.nih.gov/search/#query=C30H48O4) | 1.01 | English ivy*,*  *Fructus Akebiae,*  *Cyclocarya paliurus* | [17] |
| 19 | 475.08726 | 3.7721633 | [M − H] ^−^ | 476.09454 | Diosmetin-7-O-glucuronide | C22H20O12 | 0.94 | *Dracocephalum moldavica* | [18] |
| 20 | 559.14279 | 2.7561267 | [M − H] ^−^ | 560.15007 | Apimaysin | C27H28O13 | 1.93 | *Zea mays* | [19] |
| 21 | 577.13467 | 5.47561 | [M − H] ^−^ | 578.14194 | Procyanidin dimer | [C30H26O12](https://pubchem.ncbi.nlm.nih.gov/search/#query=C30H26O12) | 0.49 | Grape seeds, barley, hops, maize, apples, strawberries, cocoa, almonds, cinnamon, peanuts, and tea | [20] |
| 22 | 579.17198 | 3.5476033 | [M − H] ^−^ | 580.17926 | Naringerin-7-O-rutinoside (narirutin) | [C27H32O14](https://pubchem.ncbi.nlm.nih.gov/search/#query=C27H32O14) | 5.95 | Citrus fruits | [21] |
| 23 | 589.15348 | 3.00939 | [M − H] ^−^ | 590.16075 | 3'-Methoxymaysin | C28H30O14 | 2.25 | *Zea mays* | [19] |
| 24 | 593.15077 | 3.1452433 | [M − H] ^−^ | 594.15805 | Isoorientin-2''-O-rhamnoside | [C27H30O15](https://pubchem.ncbi.nlm.nih.gov/search/#collection=compounds&query_type=mf&query=C27H30O15&sort=mw&sort_dir=asc) | 0.35 | Wheat grains,  sprouts | [22] |
| 25 | 609.14595 | 3.0082467 | [M − H] ^−^ | 610.15323 | Luteolin-6,8-C-diglucoside (lucenin II) | C27H30O16 | 0.16 | Citrus fruit | [23] |
| 26 | 623.16155 | 3.2052333 | [M − H] ^−^ | 624.16882 | Diosmetin 3,8-di-C-glucoside | C28H32O16 | 0.22 | Citrus fruit | [23] |
| 27 | 739.1673 | 6.5161567 | [M − H] ^−^ | 740.17457 | Astragalin; 2'',4''-Bis-O-(4-hydroxycinnamoyl) | C39H32O15 | 0.47 | *Quercus ilex* | [24] |

**References**

1.	Götz, M.E., et al., *Myristicin and elemicin: potentially toxic alkenylbenzenes in food.* Foods, 2022. **11**(13): p. 1988.

2.	Motallebi, M., et al., *Naringenin: A potential flavonoid phytochemical for cancer therapy.* Life Sciences, 2022: p. 120752.

3.	Van Nguyen, A., et al., *Comparison of separation of seed oil triglycerides containing isomeric conjugated octadecatrienoic acid moieties by reversed-phase HPLC.* Separations, 2017. **4**(4): p. 37.

4.	Lin, Y., et al., *Luteolin, a flavonoid with potential for cancer prevention and therapy.* Current cancer drug targets, 2008. **8**(7): p. 634-646.

5.	Luo, Y., et al., *Flavanols from nature: A phytochemistry and biological activity review.* Molecules, 2022. **27**(3): p. 719.

6.	Wei, G.H., et al., *Rhizobialide: a new stearolactone produced by Mesorhizobium sp. CCNWGX022, a rhizobial endophyte from Glycyrrhiza uralensis.* Chemistry & biodiversity, 2007. **4**(5): p. 893-898.

7.	M Calderon-Montano, J., et al., *A review on the dietary flavonoid kaempferol.* Mini reviews in medicinal chemistry, 2011. **11**(4): p. 298-344.

8.	Medeiros, D.L., et al., *Rhamnetin: A review of its pharmacology and toxicity.* Journal of Pharmacy and Pharmacology, 2022. **74**(6): p. 793-799.

9.	De Marino, S., et al., *New constituents of sweet Capsicum annuum L. fruits and evaluation of their biological activity.* Journal of Agricultural and Food Chemistry, 2006. **54**(20): p. 7508-7516.

10.	Nićiforović, N. and H. Abramovič, *Sinapic acid and its derivatives: natural sources and bioactivity.* Comprehensive reviews in food science and food safety, 2014. **13**(1): p. 34-51.

11.	Wang, Y., et al., *Extraction, purification, and hydrolysis behavior of apigenin-7-O-Glucoside from Chrysanthemum morifolium tea.* Molecules, 2018. **23**(11): p. 2933.

12.	Meng, X., et al., *A kaempferol-3-O-β-d-glucoside, intervention effect of astragalin on estradiol metabolism.* Steroids, 2019. **149**: p. 108413.

13.	Pollier, J. and A. Goossens, *Oleanolic acid.* Phytochemistry, 2012. **77**: p. 10-15.

14.	Ryu, D., et al., *Luteolin-7-O-Glucuronide Improves Depression-like and Stress Coping Behaviors in Sleep Deprivation Stress Model by Activation of the BDNF Signaling.* Nutrients, 2022. **14**(16): p. 3314.

15.	Zhao, M., et al., *Determination of metabolites of diosmetin-7-O-glucoside by a newly isolated Escherichia coli from human gut using UPLC-Q-TOF/MS.* Journal of agricultural and food chemistry, 2014. **62**(47): p. 11441-11448.

16.	Mbikay, M. and M. Chrétien, *Isoquercetin as an anti-COVID-19 medication: A potential to realize.* Frontiers in Pharmacology, 2022. **13**: p. 830205.

17.	Zeng, J., et al., *Current knowledge and development of hederagenin as a promising medicinal agent: a comprehensive review.* RSC advances, 2018. **8**(43): p. 24188-24202.

18.	Wu, C., et al., *Phytochemical composition profile and space–time accumulation of secondary metabolites for Dracocephalum moldavica Linn. via UPLC–Q/TOF–MS and HPLC–DAD method.* Biomedical Chromatography, 2020. **34**(8): p. e4865.

19.	Snook, M.E., et al., *New C-4''-hydroxy derivatives of maysin and 3'-methoxymaysin isolated from corn silks (Zea mays).* Journal of Agricultural and Food Chemistry, 1995. **43**(10): p. 2740-2745.

20.	Rue, E.A., M.D. Rush, and R.B. van Breemen, *Procyanidins: A comprehensive review encompassing structure elucidation via mass spectrometry.* Phytochemistry Reviews, 2018. **17**: p. 1-16.

21.	Mitra, S., et al., *Prospective multifunctional roles and pharmacological potential of dietary flavonoid narirutin.* Biomedicine & Pharmacotherapy, 2022. **150**: p. 112932.

22.	Tomé-Sánchez, I., et al., *Soluble phenolic composition tailored by germination conditions accompany antioxidant and anti-inflammatory properties of wheat.* Antioxidants, 2020. **9**(5): p. 426.

23.	Barreca, D., et al., *Citrus flavones: An update on sources, biological functions, and health promoting properties.* Plants, 2020. **9**(3): p. 288.

24.	Wang, L.-L., et al., *Two acylated flavonoid glycosides from the leaves of Quercus dentata.* Natural Product Communications, 2010. **5**(10): p. 1934578X1000501017.
